# Computational design of chemogenetic and optogenetic split proteins

**DOI:** 10.1038/s41467-018-06531-4

**Published:** 2018-10-02

**Authors:** Onur Dagliyan, Andrey Krokhotin, Irem Ozkan-Dagliyan, Alexander Deiters, Channing J. Der, Klaus M. Hahn, Nikolay V. Dokholyan

**Affiliations:** 1 Department of Biochemistry and Biophysics, University of North Carolina at Chapel Hill, Chapel Hill, NC 27599 USA; 2 Department of Pharmacology, University of North Carolina at Chapel Hill, Chapel Hill, NC 27599 USA; 3 Lineberger Comprehensive Cancer Center, University of North Carolina at Chapel Hill, Chapel Hill, NC 27599 USA; 4 Department of Chemistry, University of Pittsburgh, Pittsburgh, PA 15260 USA; 5 Present Address: Department of Neurobiology, Harvard Medical School, Boston, MA 02115 USA; 6 Present Address: Howard Hughes Medical Institute, Stanford University School of Medicine, Stanford, CA 94305 USA; 7 Present Address: Departments of Pharmacology, and Biochemistry & Molecular Biology, Penn State College of Medicine, Hershey, PA 17033-0850 USA

## Abstract

Controlling protein activity with chemogenetics and optogenetics has proven to be powerful for testing hypotheses regarding protein function in rapid biological processes. Controlling proteins by splitting them and then rescuing their activity through inducible reassembly offers great potential to control diverse protein activities. Building split proteins has been difficult due to spontaneous assembly, difficulty in identifying appropriate split sites, and inefficient induction of effective reassembly. Here we present an automated approach to design effective split proteins regulated by a ligand or by light (SPELL). We develop a scoring function together with an engineered domain to enable reassembly of protein halves with high efficiency and with reduced spontaneous assembly. We demonstrate SPELL by applying it to proteins of various shapes and sizes in living cells. The SPELL server (spell.dokhlab.org) offers an automated prediction of split sites.

## Abstract (web summary)

Designing split protein approaches is time consuming and often results in high background activity due to spontaneous assembly. Here the authors present an automated approach which uses a split energy scoring function to identify optimal protein split sites and reduces spontaneous assembly.

## Introduction

Rapid induction of protein activity in living cells can shed light on the role of protein activation kinetics in cell physiology and can overcome the cell compensation seen with slower genetic manipulations. The specific control of protein activity in living cells has been partially achieved by modulating the location or conformation of the target protein^1–4^. The location-based control offers a broad applicability, but it often does not eliminate potential off-state background activity. The conformation-based control can be powerful to reduce the background activity, but its broad applicability has been a challenge. A strategy to control protein activity is splitting the protein and then reassembling the split halves by ligand or light-induced dimerizers^1,3,5,6^, but these methods have been prone to significant spontaneous assembly^7,8^. Moreover, the approach suffers from the difficulty of identifying effective protein split sites.

**Fig. 1:**
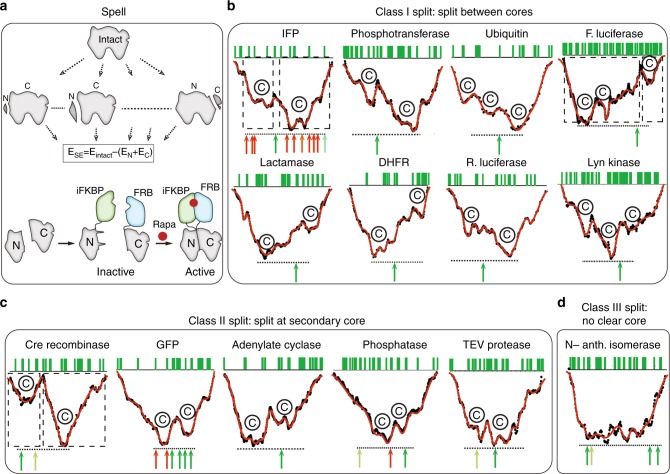
The split energy and SPELL. **a** The target protein is computationally split, and the summed energy of the split parts (N and C lobes) are subtracted from the energy of the intact protein to calculate the split energy (SE). The split energy along with other filters, including solvent accessible area (SAA) and sequence conservation, were used to identify split sites. Prevention of spontaneous assembly is achieved using insertable FKBP (iFKBP), which destabilizes one of the lobes. Rapamycin or its photoactivatable analog produces both reassembly and correct folding of the destabilized lobe. **b** Proteins that have successful split sites between cores (labeled as “C”). Arrows show split sites described in the literature. Green arrows were described as successful, red as unsuccessful. Dashed boxes indicate known domains. **c** Proteins that have successful split sites at a secondary core. **d** A protein that has no clear separation between its cores

Here, we show that split sites can be conveniently identified using a scoring function termed “split energy” with few structural parameters, and reassembly of split halves can be accomplished with minimal spontaneous assembly using an engineered domain (Fig. 1a). We demonstrate the applicability of our approach, named split proteins regulated by a ligand or by light or SPELL, on several proteins including a tyrosine kinase, guanine exchange factor, TEV protease, and guanosine nucleotide dissociation inhibitor.

## Results

### The split energy as an aid to finding split sites

Experimental trial and error-based approaches to generate split proteins often are unable to produce optimal splits with low background activity in living cells, and reassembly can be ineffective. A lack of general principles based on the mechanisms of splitting has limited broad applicability of an empirical approach to a wide range of targets. To describe rules identifying potential split sites in target proteins, we first sought to analyze existing proteins which have been split with varying degrees of effectiveness. To rationally select a potential split site on a protein structure, we first developed a physical scoring function, the “split energy”, whose minima and steep slopes identify sites where splitting should be avoided. To compute the split energy for scission at any given residue, we computed the total energies of the split parts relative to the native energy of the intact protein (Fig. 1a). The split energy profile revealed sites that are critical for protein folding, and therefore should not be used as split sites. To test the efficacy of split energy as a predictor of useful split sites, we analyzed 16 proteins with previously reported split sites (Supplementary Table 1). These analyses indicated that the split energy profile of a given protein may show either a single energy well minimum or multiwell minima. Successful split sites avoided the major split energy minima and attempts to split at the energy minima were unsuccessful (Fig. 1b–d). The split energy wells showed individual domains in the multidomain proteins firefly luciferase, IFP and Cre recombinase. In single-domain proteins, which are more challenging to split^9^, we hypothesized that energetic wells indicated “hidden subdomains” resulting from individual folding cores. Notably, these subdomains (seen in green fluorescent protein (GFP), dihydrofolate reductase (DHFR), lactamase, ubiquitin, hygromycin b phosphotransferase B, the Lyn tyrosine kinase, adenylate cyclase, PTP1B phosphatase, and TEV protease) cannot be identified using sequence-based domain databases or visual inspection of protein structure (Supplementary Figs. 1–13). A striking example is GFP, which has a single domain comprising 11 β-barrels, and an α-helix containing the covalently attached chromophore in the center, making the estimation of potential split sites by simple visual inspection of the structure challenging. The split energy profile surprisingly suggested the presence of two hidden subdomains, indicated by two separate energy wells, not detectable from the structure or contact numbers (Supplementary Fig. 1). We confirmed the presence of this multi-subdomain topology in GFP by discreet molecular dynamics simulations^10,11^. Heat-resistant residues indicated by local minima in unfolding energy diagrams suggested that the possible folding core residues are mostly located in the region between the N terminus and the loop in residues 128–133. The unfolding curve showed a sharp transition starting after this loop and reaching to the C terminus, suggesting a less stable region compared to the N-lobe, and separation of two hidden subdomains (Supplementary Fig. 1). The contact number does not accurately predict the split sites (Supplementary Fig. 1).

**Fig. 2:**
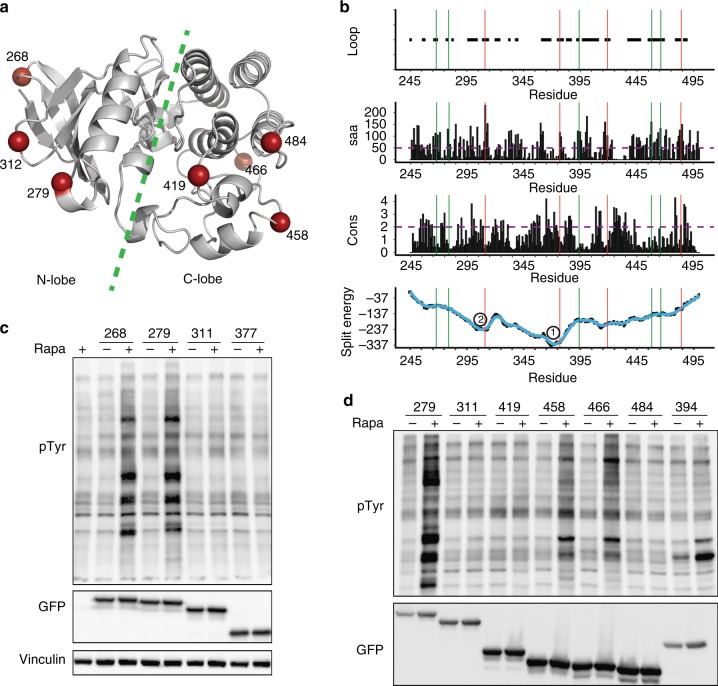
Lyn SPELL. **a** The structure of Lyn with split sites shown in red. **b** Based on the SPELL algorithm, we selected sites to test, including the promising residues 268 and 279, substantially higher in split energy than the cores at 1 and 2. 393 is a previously reported split site. **c** Phosphotyrosine blot of cell lysates with Lyn analogs split at N-lobe of the kinase domain. **d** Phosphotyrosine blot of cell lysates, including Lyn analogs split at C-lobe of the kinase domain. GFP is fused to the C terminus of Lyn to show the expression of full-length Lyn or C-lobe split protein

For the majority of benchmark proteins, successful sites were in positions between two split energy wells (Class I, Fig. 1b). In some proteins, split sites had been selected near a minor energy well (e.g., Cre recombinase, adenylate cyclase, GFP, and phosphatase, Fig. 1c, Class II). These were likely selected to reduce spontaneous folding, as was reported for Cre^12^. One exception was TEV protease, which has a split site at the major core; this split analog produced only 43% of wild-type activity^13^. One protein (N-anthranilate isomerase) did not show clearly defined cores (Class III). The split sites for this protein were close to the termini, distant from the broad region of low split energy^14^. In all three protein classes, successful split sites are at surface-exposed, evolutionarily non-conserved loops (Fig. 2b and Supplementary Figs. 1–13). Together, these observations suggested that the split energy can serve as an effective aid in finding split sites.

### The development of SPELL algorithm

We improved our algorithm by including more parameters such as solvent accessibility, sequence conservation, and loop “tightness” (Methods). For convenience, we built an open-public web server (spell.dokhlab.org) to predict split sites in a given protein (Supplementary Figure 14). We consider only surface-exposed loops with low sequence conservation. Our goal is to create an algorithm that maximizes the number of predicted true split sites while keeping the number of false-positive predictions at a minimum. The algorithm that uses split energy predicts approximately three times less potential split sites than does an algorithm solely based on solvent accessibility and sequence conservation (Supplementary Table 2). It is not feasible to directly evaluate whether this difference comes from the ability of the algorithm with the split energy to predict less false positives, because most of the literature does not report the failed split sites. However, we notice that the efficiency of both algorithms toward selecting the previously reported split sites is approximately the same (Supplementary Table 2), indicating the efficiency of split energy-based identification of split sites.

To achieve effective reassembly, we rank the loops for “tightness” (a parameter reflecting the ability of a loop to connecting two interacting structural units^15^) and by the absolute value of the split energy (Methods). The split sites with higher energy appear higher in the ranking order. This ordering is related to intrinsic limitations of the split energy profile approach. While the split energy correctly produces a first approximation of the change in free energy upon splitting, it does not take into account the entropic change, resulting in a growing number of alternative pathways through which the protein can reassemble. With a split site located deeper, more hydrophobic residues become exposed, resulting in increased spontaneous reassembly and alternative assembly pathways leading to aggregation. The majority of split sites that have been experimentally tested were ranked by our algorithm as hits in the prediction table (Supplementary Table 2). In the other cases, the loops ranked as hits by our algorithm were not previously tested experimentally.

### Prevention of spontaneous assembly with engineered FKBP12

While our algorithm can identify split sites where a protein can be successfully reconstituted, it does not eliminate the possibility that the split parts can spontaneously reassemble, or that the split parts cannot effectively reassemble upon induction. Previous studies used the dimerization of the proteins FKBP12 and FRB, driven by the small-molecule rapamycin, to induce reassembly of split proteins; FKBP12 was appended onto one-half protein and FRB onto the other. We previously showed that a version of FKBP12 missing the first 20 residues, denoted insertable FKBP12 or iFKBP, could be inserted into a protein to destabilize it until the iFKBP bound rapamycin^16–18^. For the reassembly of split proteins, we hypothesized that the use of this destabilizing version of FKBP12 could prevent spontaneous assembly, but also enable proper folding upon rapamycin-induced heterodimerization of iFKBP and FRB. Molecular dynamics simulations showed that iFKBP is substantially destabilized compared to FKBP12, which has a melting temperature of ~65 °C^19^, and the energy of iFKBP increased significantly upon refolding induced by rapamcyin (Supplementary Fig. 15). Moreover, the iFKBP N terminus is less than ~7 Å from the C terminus of FRB in the rapamycin-bound FRB–iFKBP heterodimer (Supplementary Fig. 15), so iFKBP can be readily inserted into “tight” surface loops that connect two interacting structural units. This feature provides an ability to effectively destabilize the split protein to reduce spontaneous assembly, reducing background activity, but also enables effective reassembly as the FRB terminus attached to the other split half is in close proximity to the iFKBP terminus (Supplementary Fig. 15). We tested the ability of iFKBP to prevent the spontaneous reassembly of a frequently used split protein, split-TEV^13^. To evaluate the activity of TEV analogs in living cells, we built a Förster resonance energy transfer (FRET) biosensor that produces a lower FRET signal in the presence of TEV activity (Supplementary Fig. 16). Split-TEV made using iFKBP rather than FKBP showed significantly lower activity in the absence of rapamycin. These experiments demonstrated that iFKBP can reduce spontaneous reassembly and led to an optimized split-TEV that we named TEV SPELL to denote use of the modified FKBP for optimized splitting and reassembly.

### Designing Lyn SPELL

Successful prediction of split sites in previously published split proteins suggested that we might predict split sites in new protein targets. We produced SPELL protein analogs of diverse proteins, starting with tyrosine kinase Lyn. A split analog of Lyn kinase was previously produced using the empirically identified split sites 393–394^20^. The split energy diagram of Lyn showed one major energy well (core) with two other shallow wells. Residue 393 was located in the vicinity of the core in split energy profile, so we selected residues 268 and 279, which are surface exposed and not evolutionary conserved (Fig. 2a, b). We hypothesized that this selection would make the split protein halves more stable, yet iFKBP alone would still eliminate background activity. Strikingly, in contrast to in vitro activity^20^, the published 393 site produced almost no activity in living cells upon rapamycin addition (Fig. 2c). The analogs split at 268, 279 (named Lyn SPELL), 458, and 466 had substantial activation upon rapamycin addition (Fig. 2c, d). More importantly, we selected three other non-conserved surface-exposed sites (312, 377, and 419), which are not favorable in the split energy profile, as they are in the wells. The analogs split at these sites did not produce any activity upon rapamycin addition. Furthermore, we tested the split site 484, which is both surface exposed and favorable in the split energy diagram, but is evolutionary conserved. This analog did not produce any activity in the absence or presence of rapamycin, supporting the importance of analyzing sequence conservation (Fig. 2d). In total, we successfully identify split sites that are predicted to be the top five sites by our algorithm.

### Designing GDI1 SPELL

**Fig. 3:**
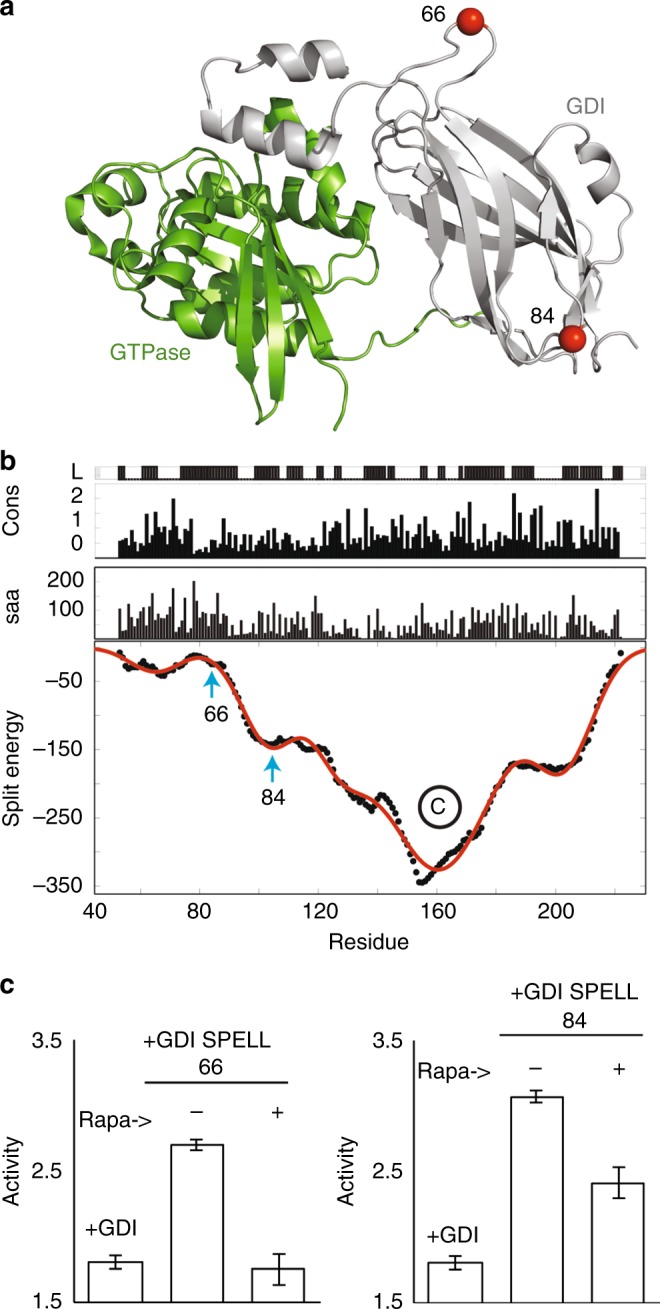
GDI SPELL. **a** The structure of RhoGDI bound to the GTPase Cdc42. **b** The SPELL algorithm indicated residue 66 as a split site. **c** The inhibitory activity of GDI SPELL (split at 66) was activated by rapamycin, whereas another design (split at 84) split at a small well did not display full activity with rapamycin. Error bars represent ± s.e.m. (*n* = 3) from three independent cell populations

We next applied SPELL to guanosine nucleotide dissociation inhibitor 1 (GDI1), a Rho GTPase family regulator which had not previously been targeted. The split energy profile indicated one major well and three shallow energy wells (Fig. 3a, b), similar to the profile of the Class III phosphatase (Fig. 1c). The first two top-ranked predictions (residues 59 and 66) are located in the same loop, so we chose to simply test residue 66. We also selected residue 84, which has an SSA of 27 Å, below the threshold defined in the algorithm. We expected that GDI split at this site would not produce activity upon reassembly (Fig. 3b). To test the efficacy of these split sites, we tested the ability of different split GDI analogs to inhibit activation of a Rac1 FRET biosensor (Rac1 FLARE DC1g^21^) in living cells. The split analog of GDI generated using residue 66 (GDI-66 SPELL) was fully activated with rapamycin, whereas the analog split at residue 84 did not provide full activity upon reassembly, as predicted (Fig. 3c).

### Designing Vav2 SPELL

**Fig. 4:**
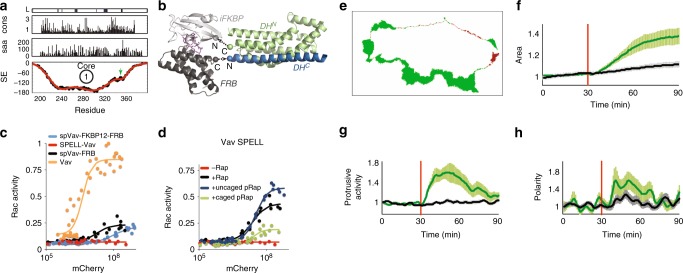
Activation of Vav2 SPELL leads to cell protrusion. **a** Split energy profile of the DH domain of Vav2. Green arrow shows the least destabilized region and chosen loop for splitting. cons = sequence conservation, saa = surface exposure. **b** A structural model of Vav2 SPELL. iFKBP (light gray) was fused to the C terminus of the N-lobe of the DH domain (green), and FRB (dark gray) was fused to the N terminus of the C-lobe of the DH domain (blue) in the presence of rapamycin (purple). **c** A dual chain Rac1 FRET sensor was used to test Vav2 analogs. The FRET ratio (reflecting the activity of Rac1) with respect to the amount of mCherry-labeled Vav2 proteins: spVav-FKBP12-FRB = split protein generated using FKBP rather than iFKBP (mCherry-DH^N^-FKBP12 and FRB-DH^C^-PH-ZnF), Vav2 SPELL (mCherry-DH^N^-iFKBP and FRB- DH^C^-PH-ZnF), spVav-FRB = split protein made with no FKBP (mCherry-DH^N^ and FRB- DH^C^-PH-ZnF), and Vav2 (mCherry-DH-PH-ZnF). **d** Vav2 SPELL activated with rapamycin or caged rapamycin, assayed as in **c**. **e** A HeLa cell expressing Vav2 SPELL, showing protrusion (green) and retraction (red) 19 min after rapamycin addition (upper left). **f**, **g**, **h** Morphology parameters (area, protrusive activity, and polarization index) of cells expressing Vav2 SPELL (green, mean ± s.e.m., *n* = 19 cells) vs. cells expressing only membrane marker (black, mean ± s.e.m., *n* = 36 cells). Rapamycin was added at 30 min (red line)

We next targeted the catalytic domain of the Rho family guanine exchange factor (GEF) Vav2, a challenging target to split as it has a single subdomain architecture, indicated by the split energy profile (Fig. 4a, b). We tested residue 347 (L4), the top prediction of our algorithm. The C-lobe fused to FRB was expressed together with either the N-lobe alone or the N-lobe fused to FKBP12. When these constructs were tested using the Rac1 biosensor, the N-lobe produced significant background activity indicating spontaneous reassembly (Fig. 4c). In contrast, no background activity was detected when iFKBP was used, further demonstrating the ability of iFKBP to destabilize the protein and prevent reassembly in the absence of rapamycin. Addition of rapamycin led to rapid and robust activation of these Vav2 SPELL analogs (Fig. 4d). Vav2 SPELL was also tested by monitoring its effects on the fluorescence spectra of cells expressing the Rac1 biosensor; the FRET emission intensity was increased upon rapamycin addition (Supplementary Fig. 17). We similarly generated a SPELL analog of intersectin-1 (ITSN), a Cdc42 GEF, with a split site at E1398, the top prediction of our algorithm. Using a Cdc42 biosensor (Cdc42 FLARE DC1g^21^), we found that ITSN SPELL was rapidly activated by rapamycin (Supplementary Fig. 18).

We showed previously that the iFKBP–FRB interaction can be induced by light using a rapamycin whose activity is blocked by a photocleavable protecting group (caged rapamycin, or pRap^15^). Using pRap, Vav2 SPELL was fully activated within 1 min of irradiation using 365 nm light (Fig. 4d). Rapamycin-mediated activation of Vav2 SPELL in HeLa cells induced protrusions within minutes, as reflected in both increased area and spreading (Fig. 4e–h and Supplementary Movie 1). Cells reached maximum protrusive activity within 10 min. This observation was consistent with Vav2′s known role in activating Rac1 to induce motility^4,22^.

## Discussion

Generation of split proteins to make biosensors and to control protein activity has been difficult due to several problems. First, it is challenging to predict an appropriate split site. Second, there can be high basal activity of split proteins in the absence of an inducer due to spontaneous protein reassembly. Third, rescued activity can be weak upon induction due to irreversible misfolding of reassembled protein or low efficiency of the inducer. To address these issues, we propose a methodology that includes (i) identification of the split sites in proteins using a novel scoring function (split energy), (ii) reduction of spontaneous assembly using an engineered domain, and (iii) effective reassembly of the split parts using a ligand or light-mediated dimerizer. We tested the accuracy of our method by predicting successful split sites on existing split proteins. We then applied our method to previously untargeted proteins such as guanosine nucleotide dissociation inhibitor 1, guanine exchange factor Vav2, and Intersectin-1, which we classified as hard targets. We further showed successful activation of SPELL Vav2 to modulate protrusion dynamics in living cells.

In summary, the split energy profile provides a clear and simple means to identify protein regions that contain viable split sites. By exploiting the destabilizing effects of iFKBP and close terminal distance of iFKBP and FRB, we greatly reduced spontaneous reassembly, yet generated effective reassembly. These approaches were used to generate five new split proteins (Lyn, GDI1, TEV, Vav2, and ITSN) subject to tight control by rapamycin or its photoactivatable analog. An online server implementing SPELL is available via http://spell.dokhlab.org.

## Methods

### DNA constructs

All restriction enzymes were purchased from New England Biolabs. Full-length (FL) Vav2 and its Dbl homology (DH), pleckstrin homology (PH), and zinc finger domain (ZnF), or DH-PH-ZnF (176–575) domains were cloned into pmCherry-C1 plasmid (Clontech) using *Bsp*eI and *Mfe*I sites. Vav2 was split as follows: Vav(FL) SPELL had the N-lobe Vav(1–346) and C-lobe Vav (348–868), whereas Vav2 (DH-PH-ZnF) SPELL had the N-lobe Vav2 (176–346) and C-lobe Vav2 (348–575). The N-lobe of either Vav2 (FL) or Vav2 (DH-PH-ZnF) was fused to the C terminus of FLAG-mCherry and cloned into the pTriex4 plasmid at the *Eco*r1 and *Not*1 sites. Either FKBP12 (ARIAD Pharmaceuticals) or iFKBP was cloned into the C terminus of the N-lobe with a short linker (GGSGGAAA) using the *Not*1 and *Xho*1 sites. Similarly, FRB (ARIAD Pharmaceuticals) was cloned into the pTriex4 plasmid at *Eco*r1 and *Not*1 sites with myc peptide at the N terminus. At the C terminus of this construct, the C-lobe of Vav2 was cloned with the same linker used in the iFKBP-Vav N-lobe construct. The same clonning strategy with the same linkers were used to generate split or human Lyn SPELL, bovine GDI1, human ITSN1, and TEV protease. In Vav2 SPELL, in order to use a single plasmid that generates a fixed stoichiometry of the split parts, the two parts (N-lobe with iFKBP and C-lobe with FRB constructs) were combined into a single pTriex4 plasmid using t2a and p2a ribozyme skipping sequences^23^. Components of the dual chain Rac1 FLARE DC1g biosensors (Ypet-PBD and dTurq-Rac1) were cloned into a single pTriex4 plasmid with t2a and p2a sequences. Cdc42 FLARE DC1g was generated similarly.

### Synthesis of photocaged rapamycin

Photocaged rapamycin was synthesized as previously described^15^.

### High-content live cell FRET assays

High-content live cell FRET imaging was performed as described previously^24^. Briefly, we seeded HEK 293T (ATCC, cat. no. CRL-3216 or cat. no. CRL-11268) cells in 96-well plates and later transfected the cells with biosensors, GEFs, or controls using the Lipofectamine and Plus reagent (Invitrogen) as suggested by the manufacturer’s manuals. The assay-generated dose–response curves to evaluate the effect of each construct at different expression levels. We used an automated microscope to image each well at CFP, YFP, FRET, and mCherry channels. We used a custom-written Matlab script for image analysis that included calculation of the sum of pixel intensities at each channel, background subtractions, bleed-through corrections, and normalization of FRET to donor ratios for each plate^24^.

### Fluorometer assays

HEK 293T (ATCC, cat. no. CRL-3216 or cat. no. CRL-11268) cells were transfected using Lipofectamine (Invitrogen) according to the manufacturer’s instructions. Twenty-four hours post transfection, cells were either treated with ethanol (vehicle) or rapamycin for 30 min. Cells were detached using trypsin, and resuspended in phosphate-buffered saline and placed in a fluorometer cuvette. The cells were analyzed using a Fluorolog SPEX 168 fluorometer. Samples were excited at 433 nm and emission was collected from 450 to 600 nm. To normalize for biosensor concentration, YFP was directly excited at 505 nm and its emission at 525 nm was measured. All measurements were normalized to CFP peak value.

### Live single-cell imaging

HeLa (ATCC) cells were plated on coverslips coated with 5 mg/mL of fibronectin (Sigma). Cells were transfected with Fugene HD (Promega) and incubated in DMEM growth medium supplemented with 10% (vol/vol) FBS at 37 °C for 24 h. L15 imaging medium (Invitrogen) supplemented with 5% (vol/vol) FBS was used for imaging. An open-heated chamber (Warner Instruments) was utilized during live cell imaging, which was performed with an Olympus IX-81 microscope equipped with an UPlanFLN ×40 objective (Pil, N.A 1.30). A Photometrics CoolSnap ES2 CCD camera (Roper Photometrics) was used to collect images. Metamorph software (Molecular Devices) was used to control the microscope and acquire images at each time point. Images were obtained at 1 min time intervals.

### Image processing

A custom-written Matlab script was used to quantify morphological changes in cells^25,26^. First, the cell boundary was detected using the MovThresh module, which automatically determines an intensity threshold for each time frame. For the images that were not detected automatically, threshold values were assigned manually. The Proactive module was used to display and quantify protrusion activity and cell area. The Squigglymorph module was used to calculate the polarity index, which was calculated by detecting protrusion and retraction using pairwise comparisons of cell boundary points.

### Kinase activity assay

HEK 293T cells (ATCC, cat. no. CRL-3216 or cat. no. CRL-11268) were transfected with an expression vector encoding Lyn using Fugene HD (Promega) and incubated in DMEM growth medium supplemented with 10% (vol/vol) FBS at 37 °C for 24 h. Cells were either treated with ethanol (vehicle) or rapamycin for 30 min, and cleared lysates were blotted with pTyr102 (Cell Signaling, cat. no. 9416 1:1000 dilution) and pTyr1000 (Cell Signaling, cat. no. 8954, 1:1000 dilution) antibodies. The uncropped scans were provided in Supplementary Fig. 19.

### Calculation of structural parameters

Solvent accessible area and secondary structure were calculated using Stride^27^. Sequences of protein families were obtained from Pfam^28^ to calculate sequence conservation. We used Kullback–Leibler conservation score to calculate the sequence conservation, and the threshold is set to 2. For the proteins that did not have structures in the protein data bank, we built homology models using I-Tasser^29^. To generate split energy profiles, target proteins were hypothetically split from residue *i*, where 1 ≤ *i* ≤ *N* and *N* is the total number of residues. For each splitting event, split energy was calculated using the equation E_N_ − (E_A_ + E_B_). The energy of native structures (E_N_) and split parts (E_A_ and E_B_) were calculated using our MEDUSA scoring function^30^. We consider a pair of consecutive residues (*i*, *i* + 1) as a potential split site if their solvent accessible area (SAA) exceeds 30 Å^2^, the Kullback–Leibler conservation score is less than 2, and at least one residue in a pair belongs to a loop region. All potential split sites within each loop are ordered by the sum of their solvent accessibilities (SSA^i^ + SSA^j^) and only the top one or two ranked sites are left for further analysis. The second top site is considered in the analysis if it is more than five residues apart from the top-ranked site. Next, we check if the selected potential split sites fall within the allowed ranges of the split energy profile. The allowed ranges are reconstructed using the following algorithm (Supplementary Fig. 14):

(A) The split energy profile is smoothed using a sliding window of size 3. The smoothing is repeated five times.
(B) The derivative of the smoothed split energy is calculated. The residues with a derivative of the smoothed split energy less than 0.5 ($\left| {E^\prime } \right| < 0.5$) are selected and the consecutive residues are grouped into intervals.
(C) Intervals from step B are iteratively extended to the residues immediately adjacent to the interval in both directions. The process is continued while the energy of the adjacent residue *E*_i_ satisfies the condition $\left| {E_i - E_{l|r}} \right| < E^{tr}$, where *E*_(*l*|*r*) is the energy of the first or last residue in the interval. *E*^*tr*^ is a threshold value. We set $E^{tr} = \left| {E^{\rm{min}}} \right|/20$, where *E*^min^ is a global energy minimum of the smoothed split energy profile. Following the extension, the overlapping intervals are merged.
(D) The intervals corresponding to minima of the energy profile are discarded. We also discard the first and last intervals if their borders include the first or the last residues of the protein.

We evaluate loop tightness, which provides a measure of how the loop bends on itself. We select the loop residues and five residues next to the first (left) and last (right) residues of the loop. These residues are required not to belong to any other loops. For each residue prior (left) to the first residue of the loop, we find the closest residue on the right. The loop is considered to be tight if the distance of at least three left-right pairs is less than 10 Å. The predicted split sites are ranked by the loop tightness and then sorted by split energy. The sites with higher split energy are reported first in the server. A predicted split site is considered to coincide with an experimentally validated split site, if predicted and experimental split sites are located within two residues.

### Generation of unfolding curves

Energies of crystal structures or homology models were first minimized using short discreet molecular dynamics simulations^31,32^ at high temperature (0.7 kcal mol^−1^ k_B_^−1^) and high heat-exchange coefficient of 10, with a harmonic potential constant of 1 kcal mol^−1^ Å^−2^. Systems were packed at low temperature (0.3 kcal mol^−1^ k_B_^−1^) with a heat-exchange coefficient of 1 and electrostatic interactions between charged residues, including acidic and basic residues. Integer charges to the central atoms of charged groups were assigned as: CZ for Arg, NZ for Lys, CG for Asp, and CD for Glu. Debye–Hückel approximation was used to model screened charge–charge interactions. By assuming a monovalent electrolyte concentration of 0.1 mM, the Debye length was set at 10 Å. Relative permittivity of water was assigned as 80. Continuous electrostatic interaction potential was discretized with an interaction range of 30 Å, where the screened potential approached zero. The simulation time for production runs of each trajectory at each temperature was ~50 ns. To identify the melting temperatures, heat capacities at constant volume (C_v_) and root mean square deviations (RMSD) at each temperature were calculated. Unfolding curves for each trajectory were generated by computing the root mean square fluctuations (RMSF) and contact number at each temperature. A simulation of each system at each temperature included ten independent trajectories.

## Electronic supplementary material

Supplementary Information

Description of Additional Supplementary Files

Supplementary Movie 1

## Data Availability

The data that support the findings of this study are available from the corresponding authors on reasonable request.

## References

[1] Spencer DM, Wandless TJ, Schreiber SL, Crabtree GR (1993). Controlling signal-transduction with synthetic ligands. Science.

[2] Wu YI (2009). A genetically encoded photoactivatable Rac controls the motility of living cells. Nature.

[3] Levskaya A, Weiner OD, Lim WA, Voigt CA (2009). Spatiotemporal control of cell signalling using a light-switchable protein interaction. Nature.

[4] Dagliyan O (2016). Engineering extrinsic disorder to control protein activity in living cells. Science.

[5] Kennedy MJ (2010). Rapid blue-light-mediated induction of protein interactions in living cells. Nat. Methods.

[6] Yazawa M, Sadaghiani AM, Hsueh B, Dolmetsch RE (2009). Induction of protein-protein interactions in live cells using light. Nat. Biotechnol..

[7] Liu Q, Tucker CL (2017). Engineering genetically-encoded tools for optogenetic control of protein activity. Curr. Opin. Chem. Biol..

[8] Kapp GT (2012). Control of protein signaling using a computationally designed GTPase/GEF orthogonal pair. Proc. Natl Acad. Sci. USA.

[9] Shekhawat SS, Ghosh I (2011). Split-protein systems: beyond binary protein-protein interactions. Curr. Opin. Chem. Biol..

[10] Ding F, Tsao D, Nie H, Dokholyan NV (2008). Ab initio folding of proteins with all-atom discrete molecular dynamics. Structure.

[11] Dagliyan O, Proctor EA, D’Auria KM, Ding F, Dokholyan NV (2011). Structural and dynamic determinants of protein-peptide recognition. Structure.

[12] Jullien N, Sampieri F, Enjalbert A, Herman JP (2003). Regulation of Cre recombinase by ligand-induced complementation of inactive fragments. Nucleic Acids Res..

[13] Wehr MC (2006). Monitoring regulated protein-protein interactions using split TEV. Nat. Methods.

[14] Paschon DE, Patel ZS, Ostermeier M (2005). Enhanced catalytic efficiency of aminoglycoside phosphotransferase (3’)-IIa achieved through protein fragmentation and reassembly. J. Mol. Biol..

[15] Karginov AV (2011). Light regulation of protein dimerization and kinase activity in living cells using photocaged rapamycin and engineered FKBP. J. Am. Chem. Soc..

[16] Karginov AV, Ding F, Kota P, Dokholyan NV, Hahn KM (2010). Engineered allosteric activation of kinases in living cells. Nat. Biotechnol..

[17] Dagliyan O (2013). Rational design of a ligand-controlled protein conformational switch. Proc. Natl Acad. Sci. USA.

[18] Dagliyan O (2017). Engineering Pak1 allosteric switches. ACS Synth. Biol..

[19] Sekiguchi M (2011). An evaluation tool for FKBP12-dependent and -independent mTOR inhibitors using a combination of FKBP-mTOR fusion protein, DSC and NMR. Protein Eng. Des. Sel..

[20] Camacho-Soto K, Castillo-Montoya J, Tye B, Ghosh I (2014). Ligand-gated split-kinases. J. Am. Chem. Soc..

[21] Machacek M (2009). Coordination of Rho GTPase activities during cell protrusion. Nature.

[22] Liu BP, Burridge K (2000). Vav2 activates Rac1, Cdc42, and RhoA downstream from growth factor receptors but not beta1 integrins. Mol. Cell. Biol..

[23] Kim JH (2011). High cleavage efficiency of a 2A peptide derived from porcine teschovirus-1 in human cell lines, zebrafish and mice. PLoS ONE.

[24] Slattery SD, Hahn KM (2014). A high-content assay for biosensor validation and for examining stimuli that affect biosensor activity. Curr. Protoc. Cell Biol..

[25] Tsygankov D (2014). CellGeo: a computational platform for the analysis of shape changes in cells with complex geometries. J. Cell Biol..

[26] Tsygankov D, Chu PH, Chen H, Elston TC, Hahn KM (2014). User-friendly tools for quantifying the dynamics of cellular morphology and intracellular protein clusters. Methods Cell Biol..

[27] Heinig M, Frishman D (2004). STRIDE: a web server for secondary structure assignment from known atomic coordinates of proteins. Nucleic Acids Res..

[28] Finn RD (2014). Pfam: the protein families database. Nucleic Acids Res..

[29] Yang J (2015). The I-TASSER Suite: protein structure and function prediction. Nat. Methods.

[30] Yin S, Ding F, Dokholyan NV (2007). Eris: an automated estimator of protein stability. Nat. Methods.

[31] Shirvanyants D, Ding F, Tsao D, Ramachandran S, Dokholyan NV (2012). Discrete molecular dynamics: an efficient and versatile simulation method for fine protein characterization. J. Phys. Chem. B.

[32] Ramachandran S, Kota P, Ding F, Dokholyan NV (2011). Automated minimization of steric clashes in protein structures. Proteins.

