# Supplementary material for: Computational design of chemogenetic and optogenetic split proteins
Source: Nat Commun. 2018 Oct 2;9:4042. doi: 10.1038/s41467-018-06531-4 (PMC6168510; doi:10.1038/s41467-018-06531-4)
Supplement: Supplementary file 1 — Supplementary Information [file 41467_2018_6531_MOESM1_ESM.pdf]

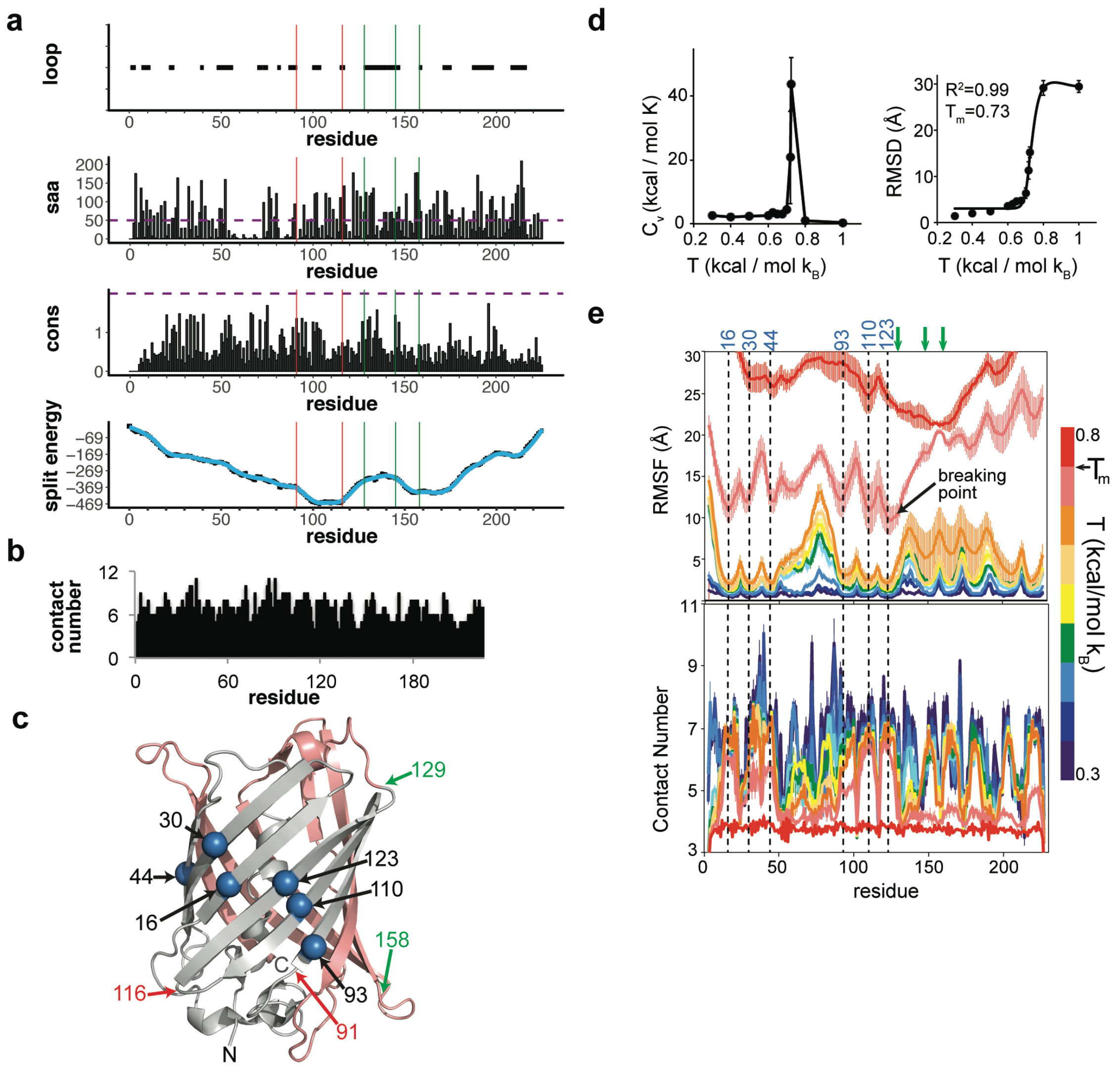

Supplementary Figure 1. Computational identification of split sites for GFP. (**a**) Loops, solvent accessible area (SAA), sequence conservation (cons), consensus of these three parameters (loop, SAA cons), and split energy. X axis = amino acid number. Green and red arrows in the split energy profile show the experimentally tested successful and unsuccessful split sites, respectively. (**b**) Number of contacts. (**c**) Structure of GFP (pdb id: 20yg) with computed core residues (blue spheres), and the experimentally tested successful (green) and unsuccessful (red) split sites. N-lobe = gray and C-lobe = salmon. (**d**) Prediction of transition temperatures based on heat capacity and average root mean square difference (RMSD) with respect to initial structure. (**e**) Root means square fluctuations (RMSF) and contact numbers of GFP at different temperatures. Green and red lines indicate experimentally tested successful and unsuccessful sites.

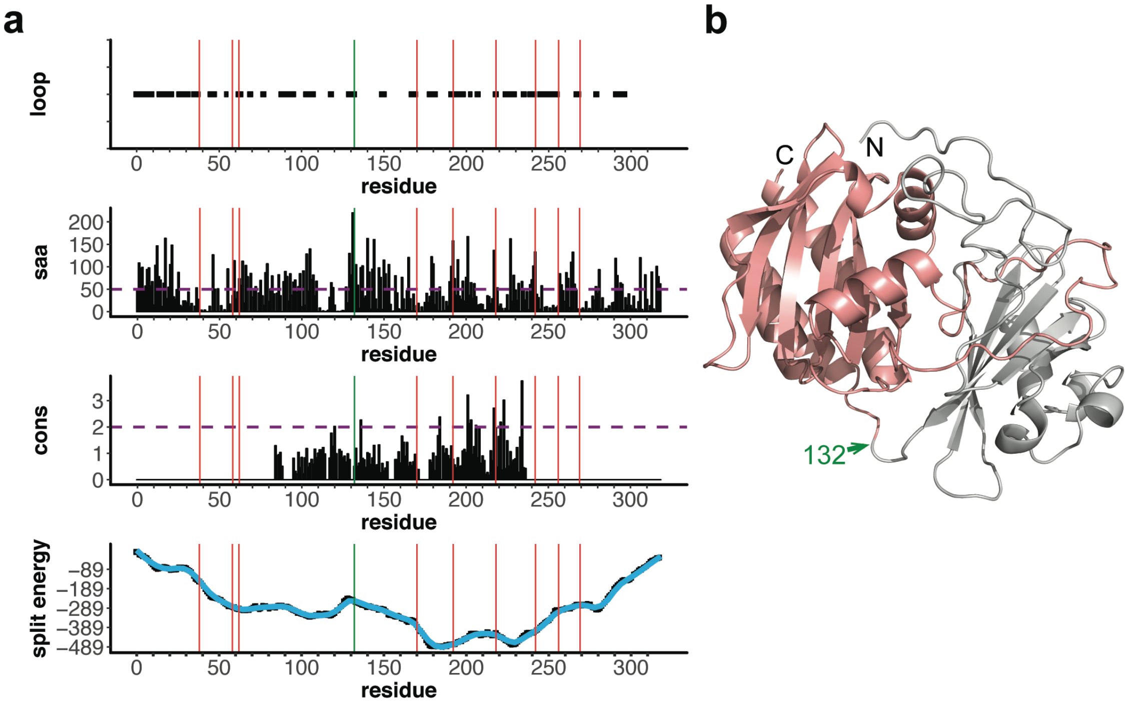

Supplementary Figure 2. Computational identification of split sites for IFP. (**a**) Loops, solvent accessible area (SAA), sequence conservation (cons), consensus of these three parameters (loop, SAA cons), and split energy. X axis = amino acid number. Green and red lines in the split energy profile show the experimentally tested successful and unsuccessful sites. (**b**) Structure of IFP (structural model was built using I-Tasser[1] based on the IFP template, pdb id: 1ztu) with the experimentally tested successful (green arrow) split sites. N-lobe = gray and C-lobe = salmon.

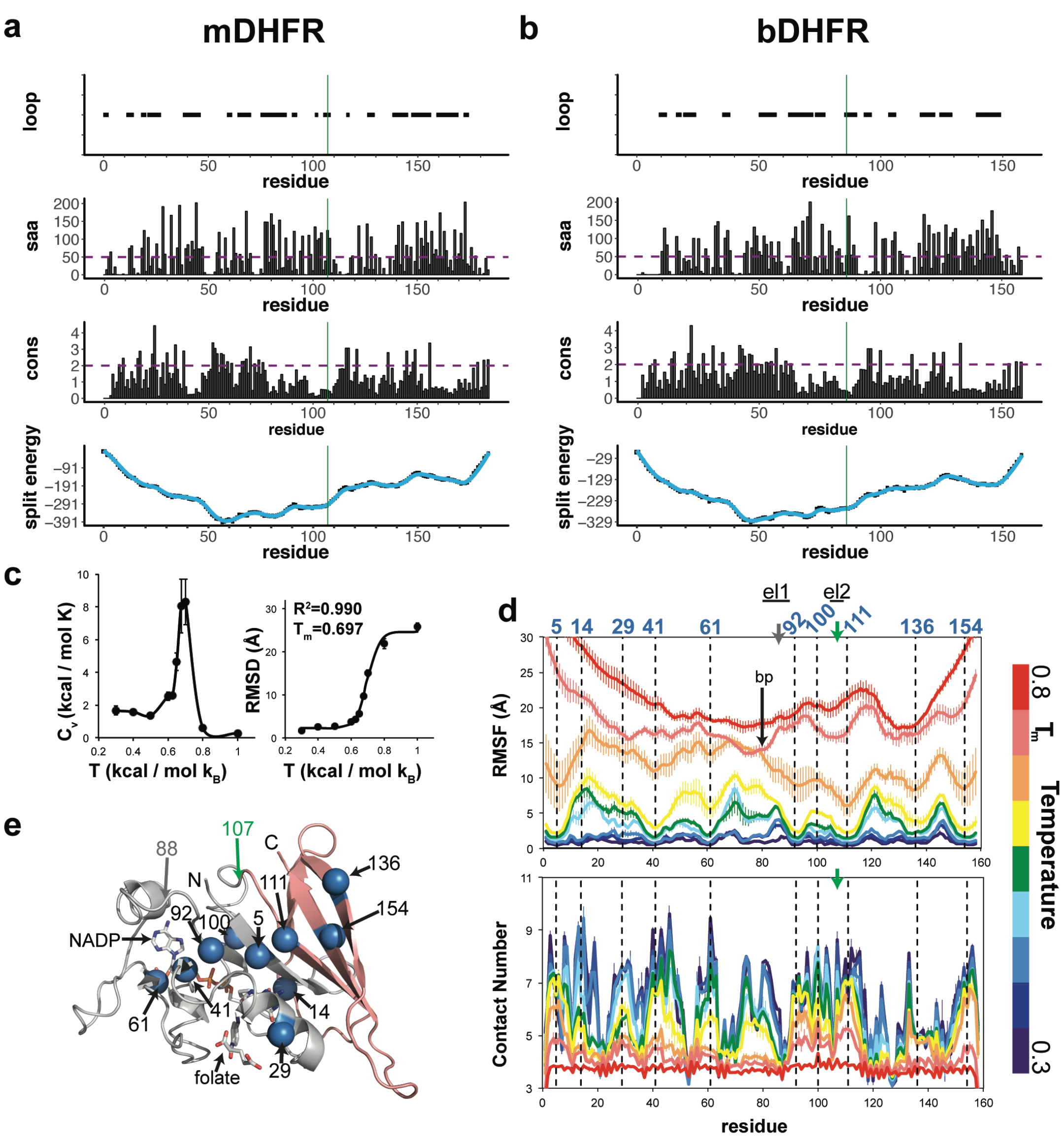

Supplementary Figure 3. Computational identification of split sites for DHFR. Loops, solvent accessible area (SAA), sequence conservation (cons), consensus of these three parameters (loop, SAA cons), and split energy of (**a**) Murine DHFR (mDHFR) and (**b**) bacterial DHFR (bDHFR). X axis = amino acid number. Green line in the split energy profile shows the experimentally tested successful split site. (**c**) Prediction of transition temperatures based on heat capacity and root mean square difference (RMSD) for bDHFR with respect to its initial structure. (**d**) Root means square fluctuations (RMSF) and contact numbers of GFP at different temperatures. Green arrow indicates experimentally tested successful site. bp: break point. (**e**) Structure of bDHFR (pdb id: 1rx2) with computed core residues (blue spheres), and the experimentally tested successful site was labeled green. Gray: N-lobe and salmon: C-lobe.

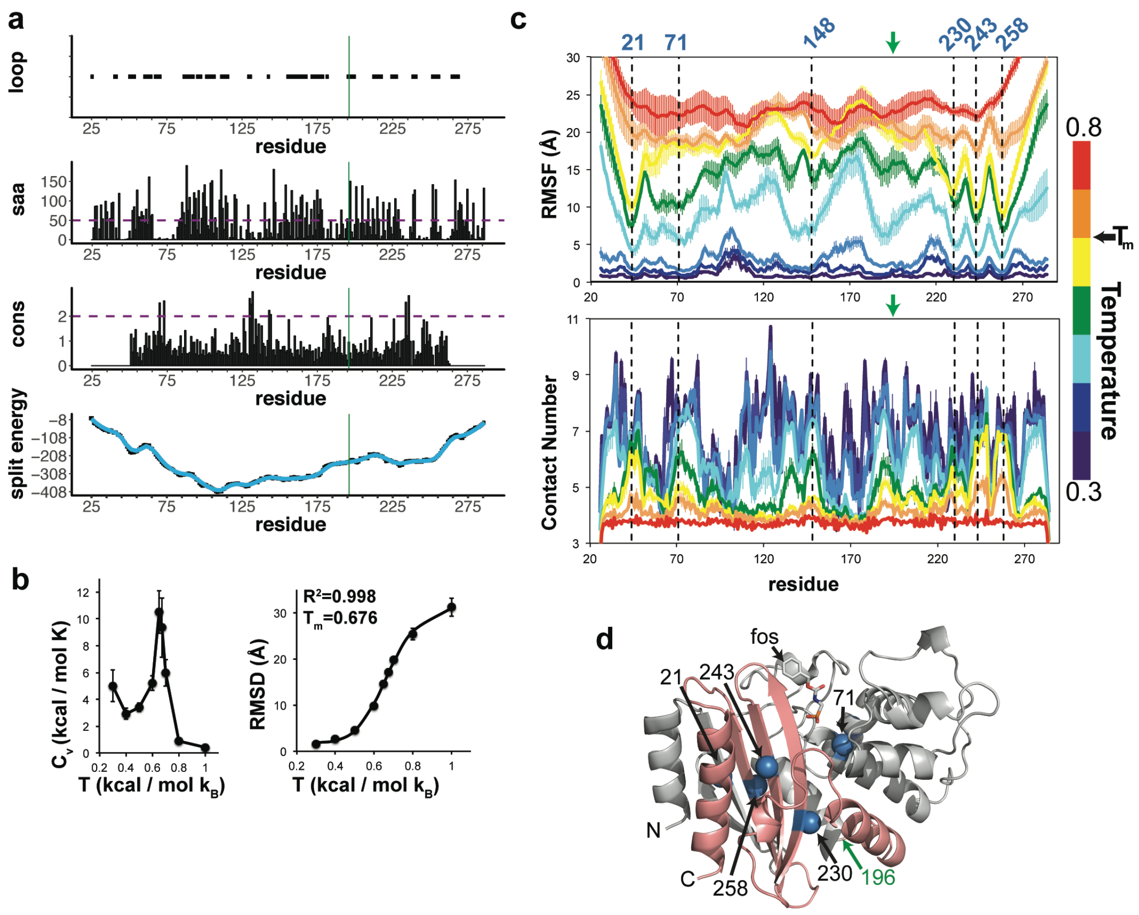

Supplementary Figure 4. Computational identification of split sites of lactamase. (**a**) Loops, solvent accessible area (SAA), sequence conservation (cons), consensus of these three parameters (loop, SAA cons), and split energy. Green line in the split energy profile shows the experimentally tested successful split site. (**b**) Prediction of transition temperatures based on heat capacity and root mean square difference (RMSD) for bDHFR. (**c**) Root means square fluctuations (RMSF) and contact numbers of GFP at different temperatures. Green arrows indicate experimentally tested successful sites. (**d**) Structure of lactamase (pdb id: 1zg4) with suggested core residues (blue spheres), and the experimentally tested successful site was labeled green. Gray: N-lobe and salmon: C-lobe.

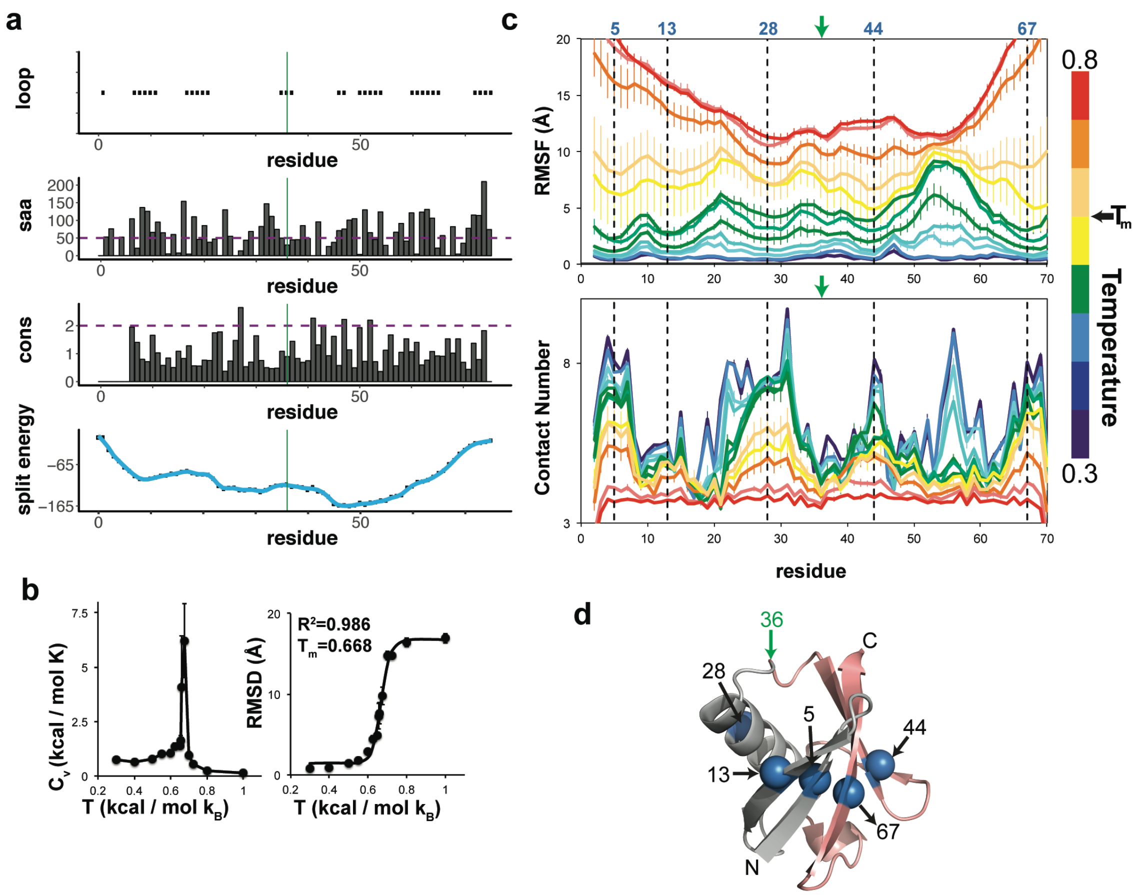

Supplementary Figure 5. Computational identification of split sites for ubiquitin. (**a**) Loops, solvent accessible area (SAA), sequence conservation (cons), consensus of these three parameters (loop, SAA cons), and split energy. Green line in the split energy profile shows the experimentally tested successful split site. (**b**) Prediction of transition temperatures based on heat capacity and root mean square difference (RMSD) for bDHFR. (**c**) Root means square fluctuations (RMSF) and contact numbers of GFP at different temperatures. Green arrow in the split energy profile shows the experimentally tested successful split sites. (**d**) Structure of ubiquitin (pdb id: 1ubq) with suggested core residues (blue spheres), and the experimentally tested successful site was labeled green. Gray: N-lobe and salmon: C-lobe.

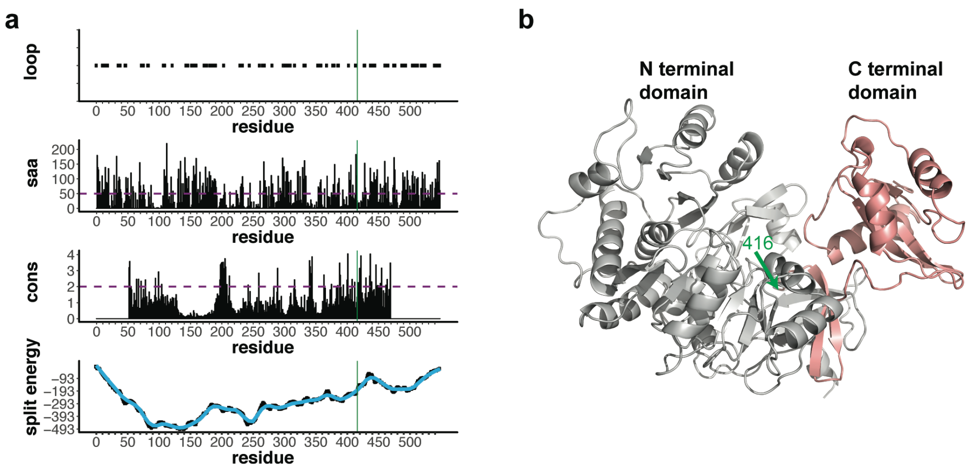

Supplementary Figure 6. Computational identification of split sites for firefly luciferase. (**a**) Loops, solvent accessible area (SAA), and split energy. Green line in the split energy profile shows the experimentally tested successful split site. (**b**) Structure of fLuciferase (structural model was made using I-Tasser[1] based on the template pdb id:1lci) with the most successful split site (green arrow). Gray: N-lobe and salmon: C-lobe.

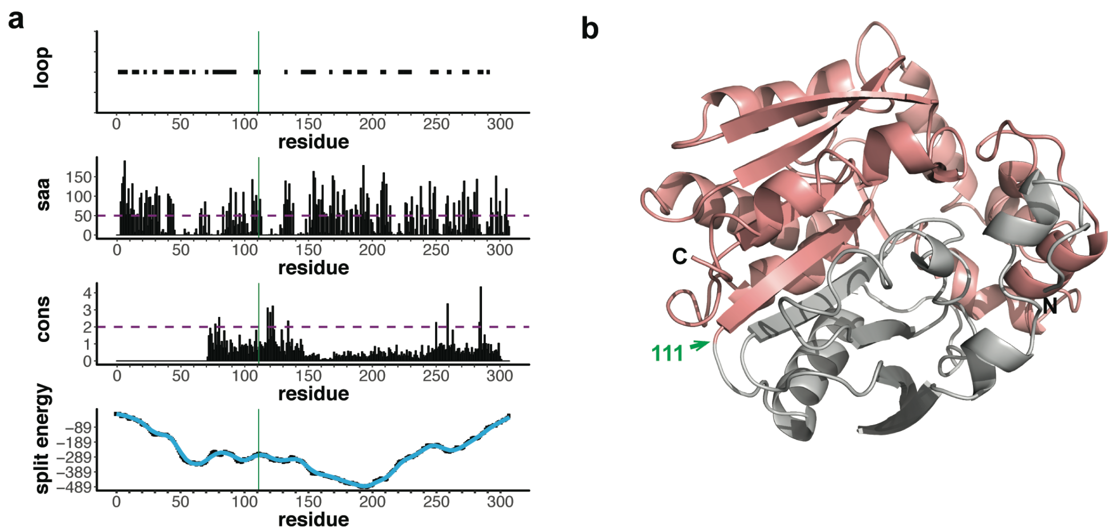

Supplementary Figure 7. Computational identification of split sites for renilla luciferase. (**a**) Loops, solvent accessible area (SAA), sequence conservation (cons), consensus of these three parameters (loop, SAA cons), and split energy. Green line in the split energy profile shows the experimentally tested successful split site. (**b**) Structure of rLuciferase (pdb id: 2psf) with the most successful split site (green arrow). Gray: N-lobe and salmon: C-lobe.

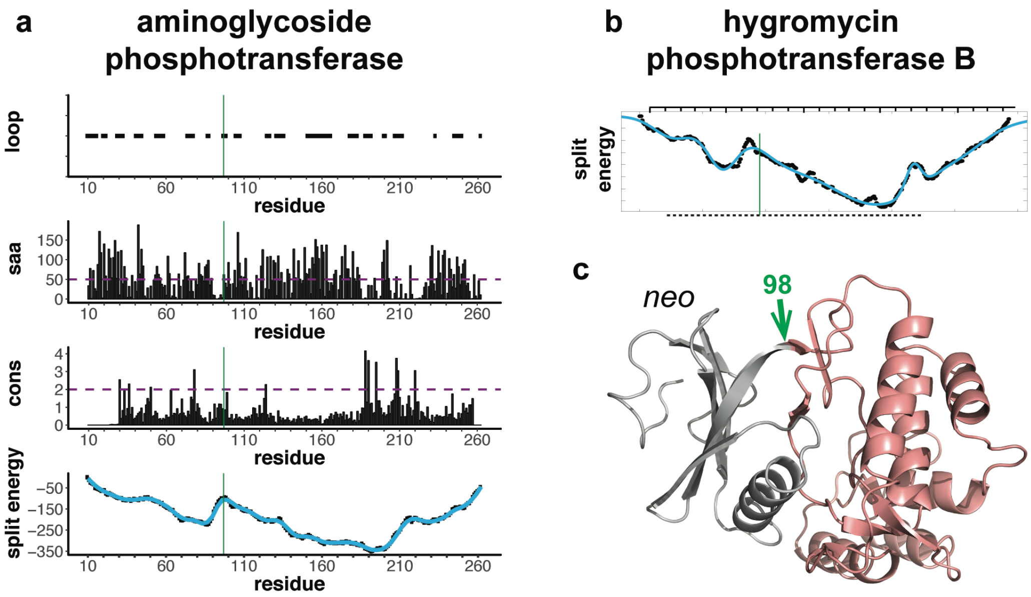

Supplementary Figure 8. Computational identification of split sites for phosphotransferase. (**a**) Loops, solvent accessible area (SAA), sequence conservation (cons), consensus of these three parameters (loop, SAA cons), and split energy of aminoglycoside phosphotransferase. (**b**) Split energy of hygromycin phosphotransferase B. Green line in the split energy profile shows the experimentally tested successful split site. (**c**) Structure of aminoglycoside phosphotransferase (pdb id: 1nd4) with the most successful split site (green arrow). Gray: N-lobe and salmon: C-lobe.

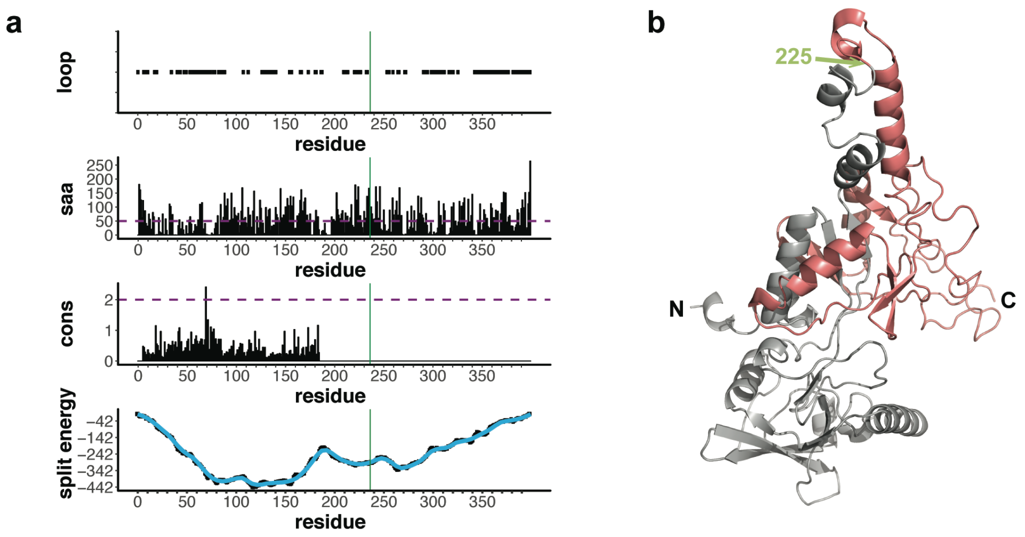

Supplementary Figure 9. Computational identification of split sites for adenylate cyclase. (**a**) Loops, solvent accessible area (SAA), sequence conservation (cons), consensus of these three parameters (loop, SAA cons), and split energy. Green line in the split energy profile shows the experimentally tested successful split site. (**b**) Structure of adenylate cyclase (structural model was made using I-Tasser[1] based on the template pdb id: 1p3j) with the most successful split site (green arrow). Gray: N-lobe and salmon: C-lobe.

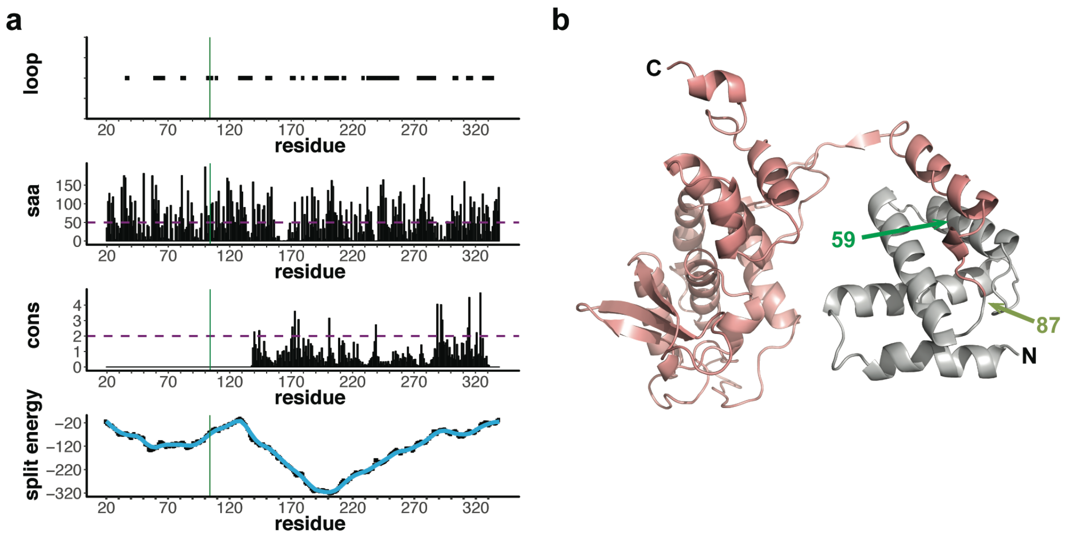

Supplementary Figure 10. Computational identification of split sites for cre recombinase. (**a**) Loops, solvent accessible area (SAA), sequence conservation (cons), and split energy. Green line in the split energy profile shows the experimentally tested primary and secondary split sites, respectively. (**b**) Structure of cre recombinase (pdb id: 3crx) with the most successful split site (green arrow). Gray: N-lobe and salmon: C-lobe.

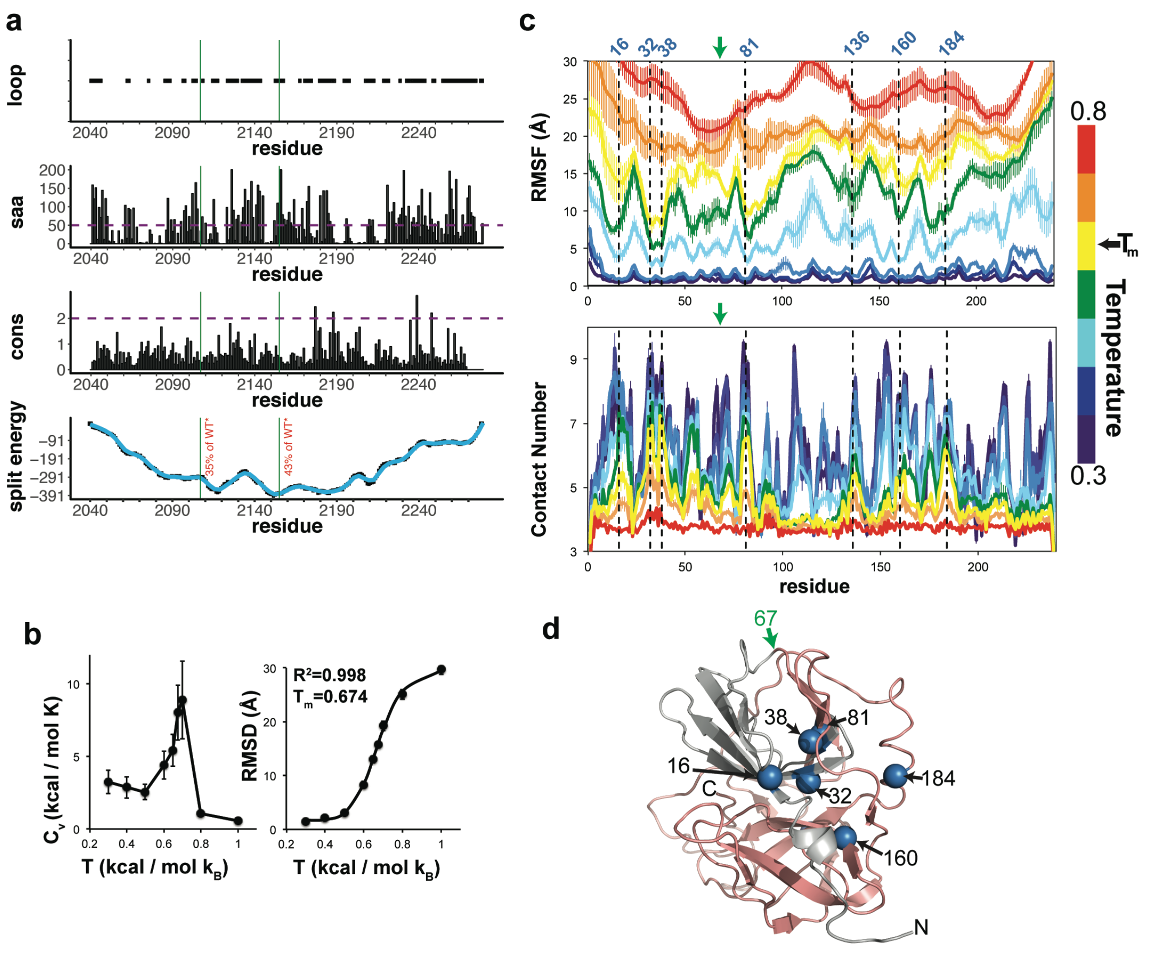

Supplementary Figure 11. Computational identification of split sites for TEV protease. (**a**) Loops, solvent accessible area (SAA), sequence conservation (cons), and split energy. Dark and light green arrows in the split energy profile show the experimentally tested primary and secondary split sites, respectively. (**b**) Prediction of transition temperatures based on heat capacity and root mean square difference (RMSD) with respect to initial structure. (**c**) Root means square fluctuations (RMSF) and contact numbers of GFP at different temperatures. Green lines indicate experimentally tested successful sites. (**d**) Structure of TEV protease (pdb id: 1q31) with the most successful split site (green arrow). Gray: N-lobe and salmon: C-lobe.

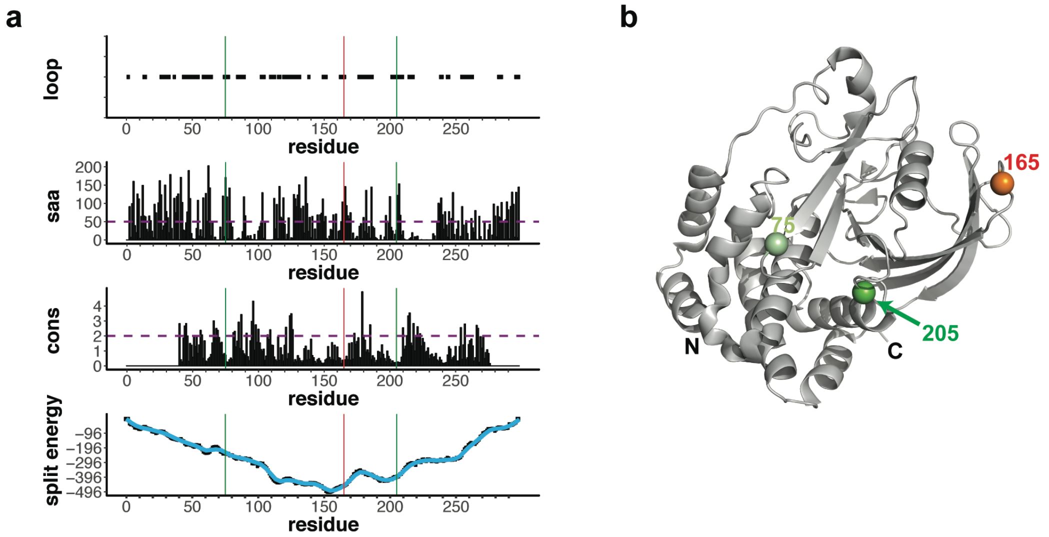

Supplementary Figure 12. Computational identification of split sites for PTP1B phosphatase. (**a**) Loops, solvent accessible area (SAA), sequence conservation (cons), consensus of these three parameters (loop, SAA cons), and split energy. Green and red lines in the split energy profile show the experimentally tested successful and unsuccessful sites. (**b**) Structure of phosphatase (pdb id: 2nt7). Gray: N-lobe and salmon: C-lobe.

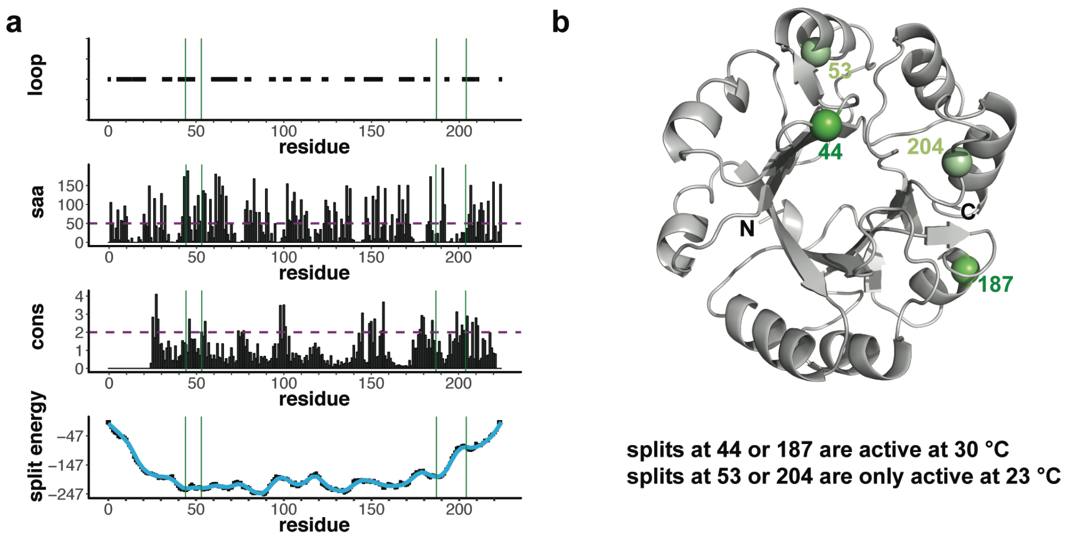

Supplementary Figure 13. Computational identification of split sites for N-anthranilate isomerase. (**a**) Loops, solvent accessible area (SAA), sequence conservation (cons), consensus of these three parameters (loop, SAA cons), and split energy. Green lines in the split energy profile show the experimentally tested split sites. Structure of anthranilate isomerase (structural model was made using I-Tasser[1] based on the template pdb id: 1lbm).

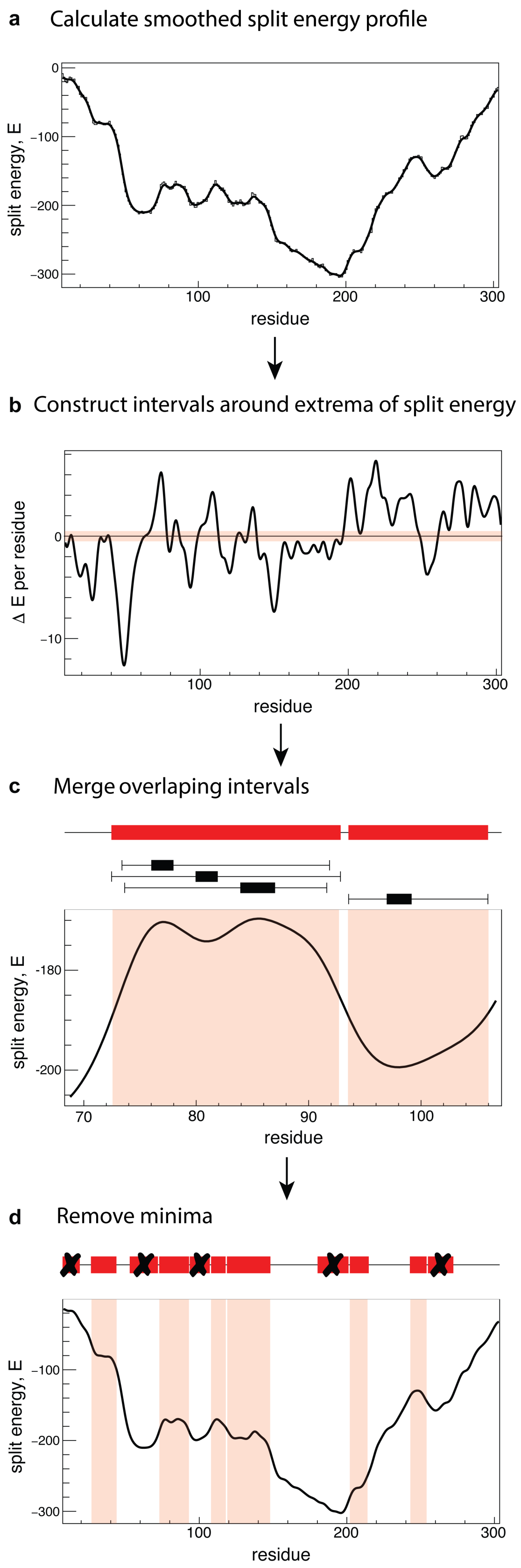
a Calculate smoothed split energy profile
split energy, E
0
−100
−200
−300
100
200
300
residue
b Construct intervals around extrema of split energy
Δ E per residue
0
−10
100
200
300
residue
c Merge overlaping intervals
split energy, E
-180
-200
70
80
90
100
residue
d Remove minima
split energy, E
−100
−200
−300
100
200
300
residue

Supplementary Figure 14. Schematic illustration of the algorithm for determination of allowed split sites based on the split energy profile (Methods). (a) Smoothed energy profile is calculated using sliding window algorithm. **(b)** A derivative of the smoothed split energy is calculated. Sites with a derivative $|E'|<0.5$ (red bar) are selected. **(c)** Sites selected at the step b (black rectangles) are used to calculate intervals around extrema of split energy (black lines). The x- axis includes only the residues between 72 and 106, which are the selected sites in the previous step. Overlapping intervals are merged (red rectangles). **(d)** The intervals corresponding to split energy minima are discarded (crossed). The first and the last intervals are discarded if their first or last residues respectively coincide with the first or last residue of a protein.

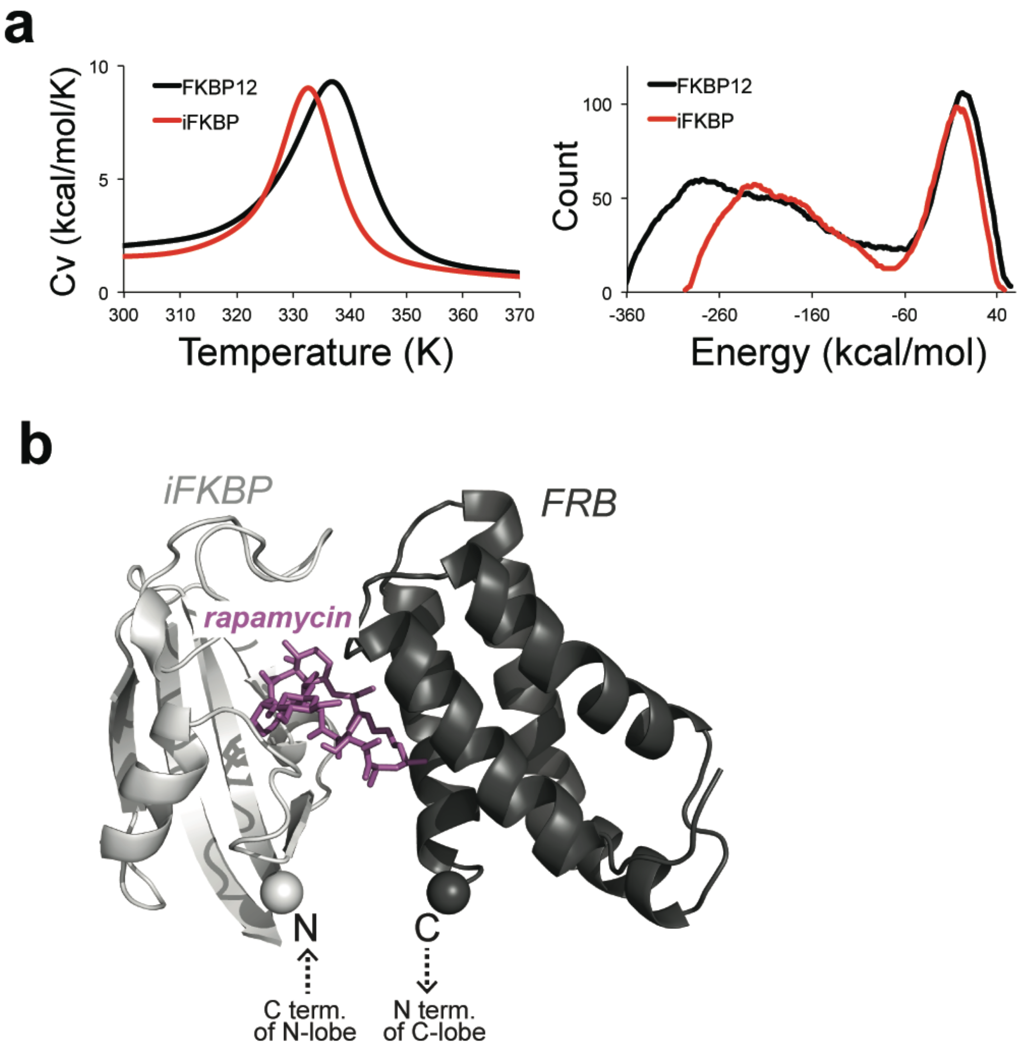

Supplementary Figure 15. Thermodynamics of FKBP12 versus iFKBP and the structural model of iFKBP with FRB and rapamycin. (**a**) Melting temperatures and energies of FKBP12 (black), and iFKBP (red). (**b**) Structural model of the iFKBP-FRB-rapamycin system. The N terminus of iFKBP was fused to the C terminus of the target protein's N-lobe, and the C terminus of the FRB was fused to the N terminus of the C-lobe of the target protein.

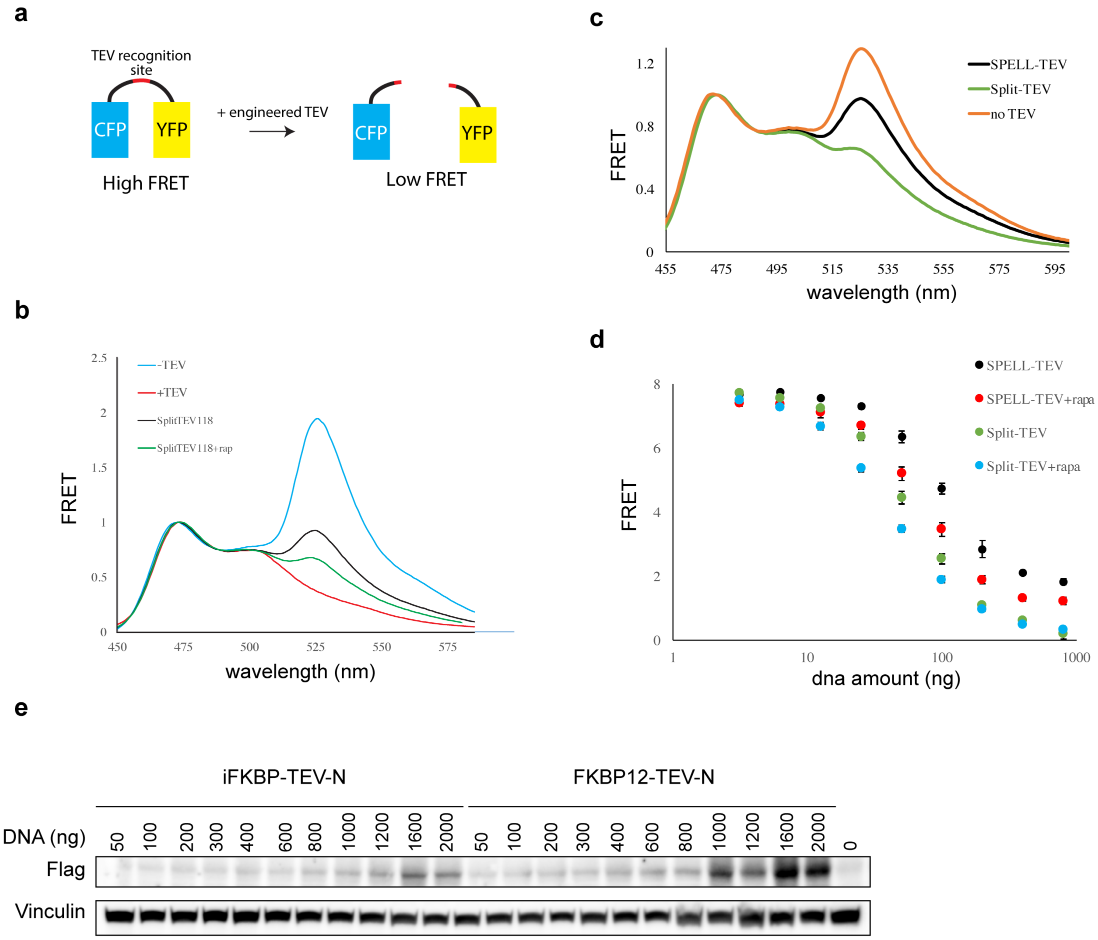

Supplementary Figure 16. A biosensor to detect TEV activity. (a) CFP and YFP were attached using a linker containing a TEV recognition site. When there was TEV activity, the linker was cleaved, resulting in lower FRET signal. **(b)** A fluorometer assay showed that SPELL TEV was significantly less active than published split TEV construct[2] in the absence of induction. **(c,d)** Titration of TEV analogues in living cells confirmed the fluorometer assay results. These cells were tested by high-content screening of live cell FRET. Error bars represent s.e.m. (n=3) from three independent cell populations. **(e)** Flag-iFKBP-TEV-N was expressed less than Flag-FKBP12-TEV-N in HEK293T cells, which were collected for immunoblotting at 24 h after transfection. Vinculin served as a loading control.

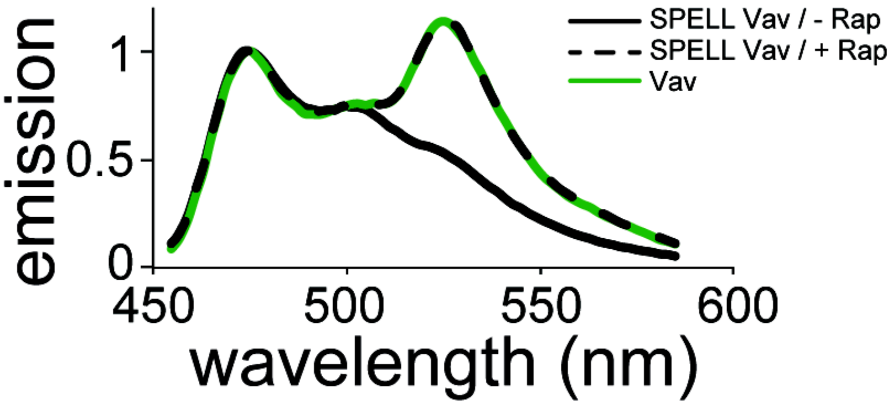

Supplementary Figure 17. Testing Split Vav2 using a fluorometer assay. SPELL Vav2 was activated upon addition of rapamycin (500 nM) to the cell media.

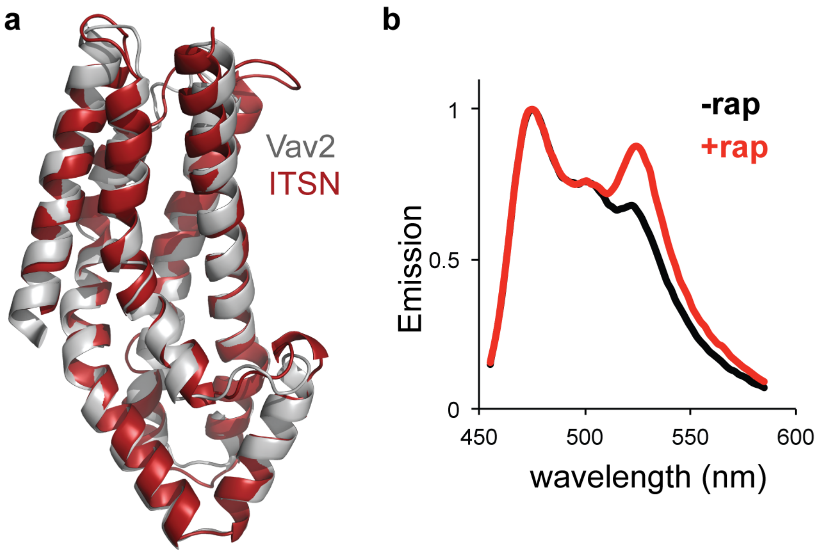

Supplementary Figure 18. Split Intersectin (ITSN). (**a**) Crystal structure of ITSN (red) and homology model of Vav2 (gray). (**b**) Normalized emission of Cdc42 sensor co-expressed with SPELL ITSN.

Figure 2c

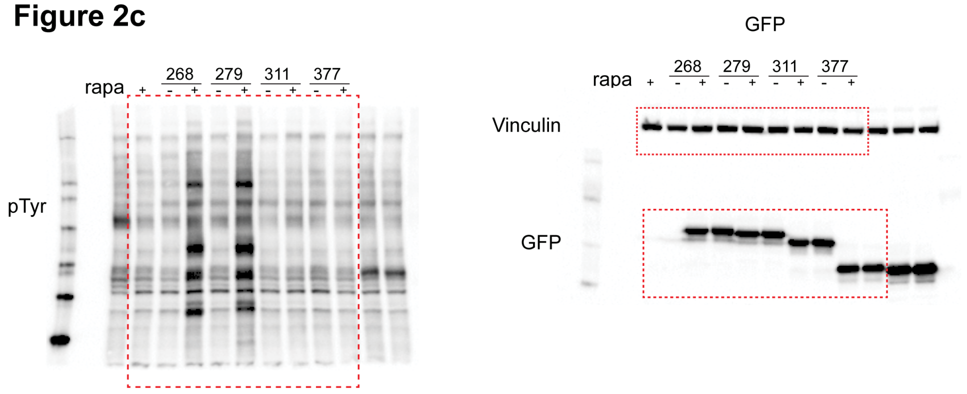

Figure 2d

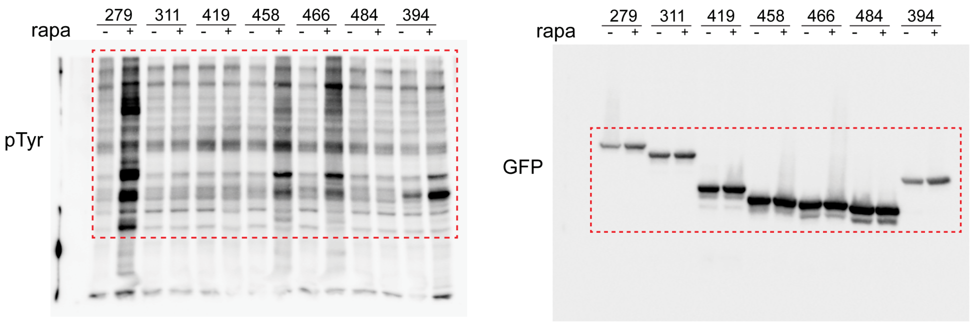

Supplementary Figure 15e

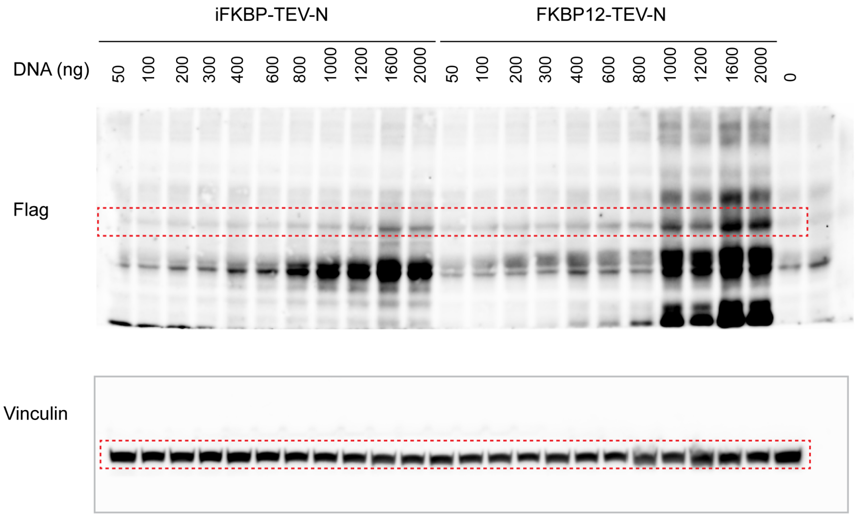

Supplementary Figure 19. Uncropped versions of the blots used in Fig. 2 and Supplementary Fig. 15. Red dashed boxes are the cropped regions.

Split protein name	Total residue	Cut site (after)	Residues In part A	Residues in part B	part A %	part B %	Reference
GFP version 1	229	157	157	72	68.6	31.4	[3]
GFP version 2	229	127	127	102	55.5	44.5	[4]
GFP version 3	229	144	144	85	62.9	37.1	[4]
GFP version 4	229	193	193	36	84.3	15.7	[5]
GFP version 5	229	212	212	17	92.6	7.4	[5]
Infrared fluorescent protein (IFP) version 1	321	132	132	189	41.1	58.9	[6]
Infrared fluorescent protein (IFP) version 2	321	252	252	69	78.5	21.5	[6]
Infrared fluorescent protein (IFP) version 3	321	293	293	28	91.3	8.7	[6]
Mammalian DHFR (mDHFR)	186	107	107	79	57.5	42.5	[7]
Bacterial DHFR (bDHFR)	159	107	107	52	67.3	32.7	[8]
Lactamase	263	171	171	92	65.0	35.0	[9]
Lyn kinase	257	148	148	109	57.6	42.4	[10]
Ubiquitin	76	36	36	40	47.4	52.6	[11]
Firefly luciferase (fLuciferase)	550	416	416	134	75.6	24.4	[12]
Renilla luciferase (rLuciferase)	307	226	226	81	73.6	26.4	[13, 14]
Aminoglycoside phosphotransferase	255	90	90	165	35.3	64.7	[15]
Hygro. phosphotransferase B	295	98	98	197	33.2	66.8	[16]
Adenylate cyclase	399	224	224	175	56.1	43.9	[17]
Cre recombinase version 1	323	41	41	282	12.7	87.3	[18]
Cre recombinase version 2	323	86	86	237	26.6	73.4	[18]
TEV protease	239	67	67	172	28.0	72.0	[2]
PTP1B phosphatase version 1	299	205	205	94	68.6	31.4	[19]
PTP1B phosphatase version 2	299	75	75	224	25.1	74.9	[19]
N-anthranilate isomerase version 1	224	44	44	180	19.6	80.4	[20]
N-anthranilate isomerase version 2	224	53	53	171	23.7	76.3	[20]
N-anthranilate isomerase version 3	224	187	187	37	83.5	16.5	[20]
N-anthranilate isomerase version 4	224	204	204	20	91.1	8.9	[20]

Supplementary Table 1. A list of split proteins.

Split protein name	Total Loops	Total validated sites [A]	Basic Selection [B]		Basic Selection [B] + Energy Profile		
			Predicted	Validated[C]	Predicted	Validated[C]	Rank[D]
Lyn kinase	21	5	18	5	11	5	1
GDI	14	2	12	2	6	2	2
Vav2	8	1	8	1	2	1	1
GFP	16	6	11	4	6	3	1
Infrared fluorescent protein (IFP)	25	3	22	2	4	2	2
Mammalian DHFR (mDHFR)	16	1	9	0	4	0	0
Bacterial DHFR (bDHFR)	13	1	6	1	2	1	2
Lactamase	18	1	16	1	4	1	3
Ubiquitin	7	1	7	1	2	1	1
Firefly luciferase (fLuciferase)	40	1	35	1	17	1	4
Renilla luciferase (rLuciferase)	23	1	17	1	6	1	5
Aminoglycoside phosphotransferase (neo)	19	1	17	1	8	1	3
Hygro. phosphotransferase B	23	1	20	1	9	1	5
Adenylate cyclase	30	1	17	1	7	1	7
Cre recombinase	17	2	15	1	7	1	1
PTP1B phosphatase	21	2	18	2	5	1	3
N-anthranilate isomerase	17	4	14	2	4	1	1
ITSN	9	2	6	2	4	2	1
TEV protease	21	1	9	0	1	0	0

[A] - This number only includes the split sites that were experimentally validated. Hence, it only provides a lower estimate of the total amount of possible split sites.
[B] – Split sites are predicted based on solvent accessibility and sequence conservation (Methods and Supplementary Fig. 14)
[C] – The experimentally validated sites that pass the selection (either Basic Selection or Basic Selection + Energy Profile)
[D] – The position of the first experimentally validated split site in ranked list of predicted split sites.

Supplementary Table 2. Efficiency benchmarking of the split site selection algorithm

Supplementary Movie 1. Vav2 SPELL in a HeLa cell. HeLa cells transfected with SPELL Vav2 were filmed for 30 min before and 60 min after addition of rapamycin. Fluorescent images were taken at one minute intervals.

References

1. Yang J, Yan R, Roy A, Xu D, Poisson J, Zhang Y. The I-TASSER Suite: protein structure and function prediction. *Nat Methods* **12**, 7-8 (2015).

2. Wehr MC*, et al.* Monitoring regulated protein-protein interactions using split TEV. *Nat Methods* **3**, 985-993 (2006).

3. Ghosh I, Hamilton AD, Regan L. Antiparallel leucine zipper-directed protein reassembly: application to the green fluorescent protein. *Journal of the American Chemical Society* **122**, 5658-5659 (2000).

4. Ito M, Ozawa T, Takada S. Folding coupled with assembly in split green fluorescent proteins studied by structure-based molecular simulations. *J Phys Chem B* **117**, 13212-13218 (2013).

5. Cabantous S*, et al.* A new protein-protein interaction sensor based on tripartite split-GFP association. *Sci Rep* **3**, 2854 (2013).

6. Tchekanda E, Sivanesan D, Michnick SW. An infrared reporter to detect spatiotemporal dynamics of protein-protein interactions. *Nat Methods* **11**, 641-644 (2014).

7. Pelletier JN, Campbell-Valois FX, Michnick SW. Oligomerization domain-directed reassembly of active dihydrofolate reductase from rationally designed fragments. *Proc Natl Acad Sci U S A* **95**, 12141-12146 (1998).

8. Remy I, Campbell-Valois FX, Michnick SW. Detection of protein-protein interactions using a simple survival protein-fragment complementation assay based on the enzyme dihydrofolate reductase. *Nat Protoc* **2**, 2120-2125 (2007).

9. Galarneau A, Primeau M, Trudeau LE, Michnick SW. Beta-lactamase protein fragment complementation assays as in vivo and in vitro sensors of protein protein interactions. *Nat Biotechnol* **20**, 619-622 (2002).

10. Camacho-Soto K, Castillo-Montoya J, Tye B, Ghosh I. Ligand-gated split-kinases. *J Am Chem Soc* **136**, 3995-4002 (2014).

11. Johnsson N, Varshavsky A. Split ubiquitin as a sensor of protein interactions in vivo. *Proc Natl Acad Sci U S A* **91**, 10340-10344 (1994).

12. Luker KE, Smith MC, Luker GD, Gammon ST, Piwnica-Worms H, Piwnica-Worms D. Kinetics of regulated protein-protein interactions revealed with firefly luciferase complementation imaging in cells and living animals. *Proc Natl Acad Sci U S A* **101**, 12288-12293 (2004).

13. Paulmurugan R, Gambhir SS. Monitoring protein-protein interactions using split synthetic renilla luciferase protein-fragment-assisted complementation. *Anal Chem* **75**, 1584-1589 (2003).

14. Hatzios SK, Ringgaard S, Davis BM, Waldor MK. Studies of dynamic protein-protein interactions in bacteria using Renilla luciferase complementation are undermined by nonspecific enzyme inhibition. *PLoS One* **7**, e43175 (2012).

15. Paschon DE, Patel ZS, Ostermeier M. Enhanced catalytic efficiency of aminoglycoside phosphotransferase (3')-IIa achieved through protein fragmentation and reassembly. *J Mol Biol* **353**, 26-37 (2005).

16. Michnick SW, Remy I, Campbell-Valois FX, Vallee-Belisle A, Pelletier JN. Detection of protein-protein interactions by protein fragment complementation strategies. *Methods Enzymol* **328**, 208-230 (2000).

17. Karimova G, Pidoux J, Ullmann A, Ladant D. A bacterial two-hybrid system based on a reconstituted signal transduction pathway. *Proc Natl Acad Sci U S A* **95**, 5752-5756 (1998).

18. Jullien N, Sampieri F, Enjalbert A, Herman JP. Regulation of Cre recombinase by ligand-induced complementation of inactive fragments. *Nucleic Acids Res* **31**, e131 (2003).

19. Camacho-Soto K, Castillo-Montoya J, Tye B, Ogunleye LO, Ghosh I. Small molecule gated split-tyrosine phosphatases and orthogonal split-tyrosine kinases. *J Am Chem Soc* **136**, 17078-17086 (2014).

20. Tafelmeyer P, Johnsson N, Johnsson K. Transforming a (beta/alpha)8--barrel enzyme into a split-protein sensor through directed evolution. *Chem Biol* **11**, 681-689 (2004).
