# Supplementary material for: Computational design of chemogenetic and optogenetic split proteins
Source: Nat Commun. 2018 Oct 2;9:4042. doi: 10.1038/s41467-018-06531-4 (PMC6168510; doi:10.1038/s41467-018-06531-4)
Supplement: Supplementary file 2 — Description of Additional Supplementary Files [file 41467_2018_6531_MOESM2_ESM.pdf]

Description of Additional Supplementary Files

File Name: Supplementary Movie 1
Description: **Vav2 SPELL in a HeLa cell.** HeLa cells transfected with SPELL Vav2 were filmed for 30 min before and 60 min after addition of rapamycin. Fluorescent images were taken at one minute intervals.
